# Transcriptomic and epigenetic dissection of spinal ependymoma (SP-EPN) identifies clinically relevant subtypes enriched for tumors with and without *NF2* mutation

**DOI:** 10.1007/s00401-023-02668-9

**Published:** 2024-01-24

**Authors:** Sina Neyazi, Erika Yamazawa, Karoline Hack, Shota Tanaka, Genta Nagae, Catena Kresbach, Takayoshi Umeda, Alicia Eckhardt, Kenji Tatsuno, Lara Pohl, Taijun Hana, Michael Bockmayr, Phyo Kim, Mario M. Dorostkar, Toshihiro Takami, Denise Obrecht, Keisuke Takai, Abigail K. Suwala, Takashi Komori, Shweta Godbole, Annika K. Wefers, Ryohei Otani, Julia E. Neumann, Fumi Higuchi, Leonille Schweizer, Yuta Nakanishi, Camelia-Maria Monoranu, Hirokazu Takami, Lara Engertsberger, Keisuke Yamada, Viktoria Ruf, Masashi Nomura, Theresa Mohme, Akitake Mukasa, Jochen Herms, Shunsaku Takayanagi, Martin Mynarek, Reiko Matsuura, Katrin Lamszus, Kazuhiko Ishii, Lan Kluwe, Hideaki Imai, Andreas von Deimling, Tsukasa Koike, Martin Benesch, Yoshihiro Kushihara, Matija Snuderl, Shohei Nambu, Stephan Frank, Takaki Omura, Christian Hagel, Kazuha Kugasawa, Viktor F. Mautner, Koichi Ichimura, Stefan Rutkowski, Hiroyuki Aburatani, Nobuhito Saito, Ulrich Schüller

**Affiliations:** 1 Department of Pediatric Hematology and Oncology, University Medical Center Hamburg-Eppendorf, Hamburg, Germany; 2 Research Institute Children’s Cancer Center Hamburg, Hamburg, Germany; 3 Department of Neurosurgery, Graduate School of Medicine, The University of Tokyo, Tokyo, Japan; 4 Genome Science and Medicine Laboratory, Research Center for Advanced Science and Technology, The University of Tokyo, Tokyo, Japan; 5 Institute of Neuropathology, University Medical Center Hamburg-Eppendorf, Hamburg, Germany; 6 Mildred Scheel Cancer Career Center HaTriCS4, University Medical Center Hamburg-Eppendorf, Hamburg, Germany; 7 Department of Radiotherapy and Radiation Oncology, Hubertus Wald Tumor Center, University Cancer Center Hamburg, University Medical Center Hamburg-Eppendorf, Hamburg, Germany; 8 Utsunomiya Neurospine Center, Symphony Clinic, Utsunomiya, Japan; 9 Center for Neuropathology and Prion Research, Faculty of Medicine, Ludwig-Maximilians-Universität Munich, Munich, Germany; 10 German Center for Neurodegenerative Diseases, Munich, Germany; 11 Department of Neurosurgery, Osaka Medical and Pharmaceutical University, Osaka, Japan; 12 Department of Neurosurgery, Tokyo Metropolitan Neurological Hospital, Tokyo, Japan; 13 Department of Neuropathology, Institute of Pathology, University of Heidelberg, Heidelberg, Germany; 14 Clinical Cooperation Unit Neuropathology, German Cancer Research Center (DKFZ), German Consortium for Translational Cancer Research (DKTK), Heidelberg, Germany; 15 Department of Laboratory Medicine and Pathology, Tokyo Metropolitan Neurological Hospital, Tokyo, Japan; 16 Center for Molecular Neurobiology Hamburg, University Medical Center Hamburg-Eppendorf, Hamburg, Germany; 17 Department of Neurosurgery, Tokyo Metropolitan Cancer and Infectious Diseases Center Komagome Hospital, Tokyo, Japan; 18 Department of Neurosurgery, University of Teikyo Hospital, 2-11-1 Kaga, Itabashi-ku, Tokyo Japan; 19 Institute of Neurology (Edinger Institute), University Hospital Frankfurt, Goethe University, Frankfurt Am Main, Germany; 20 German Cancer Research Center (DKFZ), German Cancer Consortium (DKTK), Partner Site Frankfurt/Mainz, Frankfurt Am Main, Germany; 21 Frankfurt Cancer Institute (FCI), Frankfurt Am Main, Germany; 22 Department of Neurosurgery, Osaka Metropolitan City University Graduate School of Medicine, Osaka, Japan; 23 Department of Neuropathology, Institute of Pathology, University of Würzburg, Würzburg, Germany; 24 Division of Pediatric Hematology and Oncology, Department of Pediatrics and Adolescent Medicine, Medical University of Graz, Graz, Austria; 25 Department of Neurosurgery, University Medical Center Hamburg-Eppendorf, Hamburg, Germany; 26 Department of Neurosurgery, Graduate School of Medical Sciences, Kumamoto University, Kumamoto, Japan; 27 Department of Neurology, University Medical Center Hamburg-Eppendorf, Hamburg, Germany; 28 Department of Neurosurgery, Japan Community Health Care Organization Tokyo Shinjuku Medical Center, Tokyo, Japan; 29 Department of Pathology, NYU Langone Health, New York City, USA; 30 Division of Neuropathology, Institute of Medical Genetics and Pathology, University Hospital Basel, University of Basel, Basel, Switzerland; 31 Department of Brain Disease Translational Research, Juntendo University Graduate School of Medicine, Bunkyo-Ku, Tokyo, Japan

**Keywords:** Ependymoma, Classification, *NF2*-related schwannomatosis, Transcriptomics, DNA methylation

## Abstract

Ependymomas encompass multiple clinically relevant tumor types based on localization and molecular profiles. Tumors of the methylation class “spinal ependymoma” (SP-EPN) represent the most common intramedullary neoplasms in children and adults. However, their developmental origin is ill-defined, molecular data are scarce, and the potential heterogeneity within SP-EPN remains unexplored. The only known recurrent genetic events in SP-EPN are loss of chromosome 22q and *NF2* mutations, but neither types and frequency of these alterations nor their clinical relevance have been described in a large, epigenetically defined series. Transcriptomic (*n* = 72), epigenetic (*n* = 225), genetic (*n* = 134), and clinical data (*n* = 112) were integrated for a detailed molecular overview on SP-EPN. Additionally, we mapped SP-EPN transcriptomes to developmental atlases of the developing and adult spinal cord to uncover potential developmental origins of these tumors. The integration of transcriptomic ependymoma data with single-cell atlases of the spinal cord revealed that SP-EPN display the highest similarities to mature adult ependymal cells. Unsupervised hierarchical clustering of transcriptomic data together with integrated analysis of methylation profiles identified two molecular SP-EPN subtypes. Subtype A tumors primarily carried previously known germline or sporadic *NF2* mutations together with 22q loss (bi-allelic *NF2* loss), resulting in decreased *NF2* expression. Furthermore, they more often presented as multilocular disease and demonstrated a significantly reduced progression-free survival as compared to SP-EP subtype B. In contrast, subtype B predominantly contained samples without *NF2* mutation detected in sequencing together with 22q loss (monoallelic *NF2* loss). These tumors showed regular *NF2* expression but more extensive global copy number alterations. Based on integrated molecular profiling of a large multi-center cohort, we identified two distinct SP-EPN subtypes with important implications for genetic counseling, patient surveillance, and drug development priorities.

**Supplementary Information:**

The online version contains supplementary material available at 10.1007/s00401-023-02668-9.

## Introduction

Ependymomas are a heterogeneous group of primary central nervous system tumors, affecting pediatric and adult patients and occurring along the entire neural axis [36]. Recent comprehensive studies identified distinct epigenetic and transcriptional profiles and biological markers across ependymal tumors, resulting in an updated molecular classification comprising ten ependymoma types [15, 36] recognized by the 2021 WHO central nervous system (CNS) tumor classification [27, 31]. Subsequent studies have revealed further intertumoral heterogeneity within these types, resulting in a granular distinction of several clinically relevant molecular subtypes [6, 10, 35]. Among the ten molecular ependymoma types, spinal ependymomas (SP-EPN) are one of four tumor types located in the spinal cord: Subependymoma (SE), myxopapillary ependymoma (MPE), spinal ependymoma with *MYCN* amplification (SP-MYCN), and spinal ependymoma (SP-EPN) [31].

SP-EPN are defined by their spinal localization, a distinct DNA methylation profile, and the absence of both morphological features of MPE or SE as well as *MYCN* amplification [31]. Studies of limited SP-EPN cohorts indicate that histologically, these tumors are mostly low-grade and occur predominantly in adults [36, 49]. The only known recurrent genetic events in SP-EPN are *NF2* mutations and loss of chromosomal arm 22q, which harbors the *NF2* gene [36, 49]. However, data on the frequency of *NF2* mutations vary and originate from small series lacking epigenetic or transcriptomic characterization [5, 13, 28, 57]. *NF2* mutations associated with SP-EPN usually occur either as germline or mosaic mutations in patients with *NF2*-related schwannomatosis (NF2), previously known as neurofibromatosis type 2 [38], or as somatic mutations in patients without NF2 [26, 39, 42, 47]. *NF2*-related schwannomatosis is a tumor predisposition syndrome caused by loss-of-function alterations in *NF2* resulting in the development of schwannomas, meningiomas, and ependymomas. Clinical severity of NF2 depends on the type and position of the *NF2* alteration, with ependymomas and meningiomas more often arising in patients with truncating *NF2* mutations compared to milder clinical phenotypes in patients with missense or mosaic mutations [11, 19].

Clinically, ependymomas of the spinal cord are the most common intramedullary spinal tumors and account for significant morbidity in children and adults [34]. Even though overall survival of patients with spinal ependymomas is generally good, functional outcome and risk of recurrence are associated with the timing and extent of treatment [14]. Gross total surgical resection, sometimes followed by radiotherapy, is the current standard of care for spinal ependymomas [41]. However, it is accompanied by the risk of postoperative complications due to critical location and infiltrative tumor growth [1]. To develop novel treatment strategies for SP-EPN, detailed insights into the molecular landscape of these tumors are urgently needed.

Here, we utilized epigenetic, transcriptomic, and genomic approaches to profile a large cohort of SP-EPN. We integrated newly defined molecular signatures with clinical data, potentially aiding in the molecular stratification and identification of patients at higher risk of progression.

## Methods

**Fig. 1:**
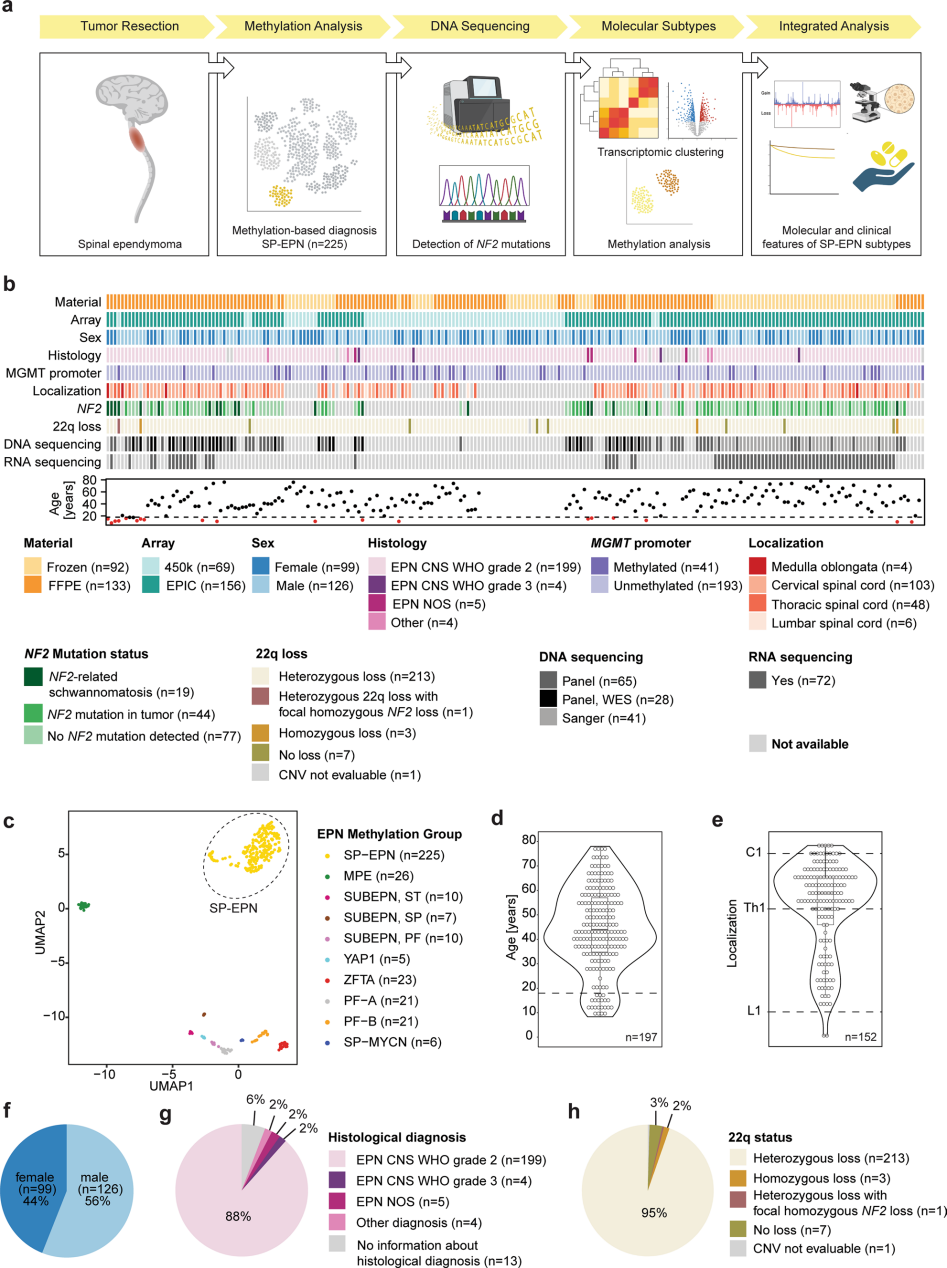
Clinical and molecular characteristics of the SP-EPN cohort. **a** Schematic of the workflow: After surgical resection of spinal ependymal tumors, 225 samples were included in this cohort based on their assignment to the methylation class “ependymoma, spinal” (SP-EPN) by the “Heidelberg CNS tumor classifier”. SP-EPN samples underwent next generation targeted panel sequencing or Sanger sequencing to detect mutations in *NF2*. Next, RNA sequencing of a subset of samples identified two distinct transcriptional SP-EPN subgroups that were then integrated with an extended methylation data set. Subsequent molecular, histopathological, and clinical analyses revealed further differences between the two molecular SP-EPN subgroups. **b** Clinico-molecular cohort characteristics (*n* = 225) and applied analysis methods. **c** UMAP of 225 SP-EPN cases clustering apart from other molecular ependymoma types. **d** Violin plot representing the age distribution of patients in the SP-EPN cohort (*n* = 197). Underlying dot plot showing the age of each individual patient. Underlying box plot displaying the first to the third quartile of the age distribution with a line in the middle representing the median value. Dotted line representing the cutoff for pediatric vs adult patients (18 years). **e** Violin plot showing the anatomical localization of tumors in the SP-EPN cohort (*n* = 152). Underlying dot plot showing the spinal localization of each individual patient. Underlying box plot displaying the first to the third quartile of the location distribution with a line in the middle representing the median value. Dotted lines representing the anatomical distinction between cervical (C1), thoracic (Th1) and lumbar (L1) spinal cord. **f** Pie chart with representation of the sex distribution of patients in the SP-EPN cohort (*n* = 225). **g** Pie chart showing the histological diagnosis of SP-EPN cases (*n* = 225). **h** Pie chart depicting the 22q status of the SP-EPN cases based on methylation data (*n* = 224; for one case of the cohort, CNV were not evaluable due to low quality). *CNS* central nervous system, *NOS *not otherwise specified, *WES* whole exome sequencing, *MPE* myxopapillary ependymoma, *SUBEPN *subependymoma, *PF *posterior fossa, *CNV* copy number variations

The multicenter study cohort (*n* = 225) consists of 170 unpublished and 55 previously published SP-EPN cases. All samples were included based on global DNA methylation profiling with a score of ≥ 0.9 for the methylation class “ependymoma, spinal” according to the “Heidelberg” methylation profiling classifier (www.molecularneuropathology.org, version v12.5; Suppl. Table 1). For unpublished cases, tumor samples and de-identified clinical data were collected in accordance with local ethical rules for the use of patient material. For published cases, raw methylation data and de-identified clinical data were obtained from Gene Expression Omnibus (GEO) and the corresponding authors [9, 36, 49]. For the comparison of SP-EPN with other molecular ependymoma types (Fig. 1c), raw methylation data were obtained from previously published data sets [6, 9, 10, 15, 35, 36].

Further methods for sample processing and data analysis are described in the supplementary methods section.

## Results

### Clinical and pathological features of SP-EPN

In this multi-institutional study, we conducted integrated profiling of molecular, histological, and clinical features of 225 spinal ependymomas, selected only by assignment to the methylation class “ependymoma, spinal” (SP-EPN) by the “Heidelberg CNS tumor classifier” (Fig. 1a–b; Suppl. Table 1). SP-EPN presented with variable clinicopathological features, as summarized in Fig. 1b and described in detail below. Global methylation analysis confirmed that all cases of this cohort were clearly distinguishable from other ependymal tumors [9, 10, 15, 35, 36] (total *n* = 354), including those occurring in the spinal cord, such as MPE or SP-MYCN (Fig. 1c).

Median age at resection was 44 years, ranging from 8.3 to 78 years and encompassing 21 pediatric (< 18 years) and 176 adult patients (Fig. 1b, d). The cohort included spinal tumors spanning from the medulla oblongata to lumbar segments, with most tumors located in the lower cervical spinal cord (median localization: C7; Fig. 1b, e). Samples were obtained from 126 male and 99 female patients, indicating a slight male predilection (Fig. 1b, f). The predominant initial histopathological diagnosis was CNS WHO grade 2 ependymoma (*n* = 199). Still, few cases were assigned to other histological diagnoses including spinal ependymoma, not otherwise specified (SP-EPN NOS, *n* = 5), CNS WHO grade 3 ependymoma (*n* = 4), spinal subependymoma CNS WHO grade 1 (*n* = 1), low-grade glioma (*n* = 1), glial tumor (*n* = 1), and pilocytic astrocytoma (*n* = 1; Fig. 1b and g; Suppl. Table 1). Consistent with previous findings [36, 49], chromosomal loss of 22q, as inferred from DNA methylation data, was detected in the majority of SP-EPN (97%, *n* = 217; for one case of the cohort, CNV were not evaluable due to low quality; Fig. 1b and h). Among these, 213 cases (98%) exhibited heterozygous 22q loss, while only three cases (1%) displayed homozygous 22q loss (Suppl. Figure 1a–c) and one additional case showed heterozygous 22q loss together with focal homozygous *NF2* loss (Suppl. Figure 1d).

### SP-EPN present with and without NF2 mutations

**Fig. 2:**
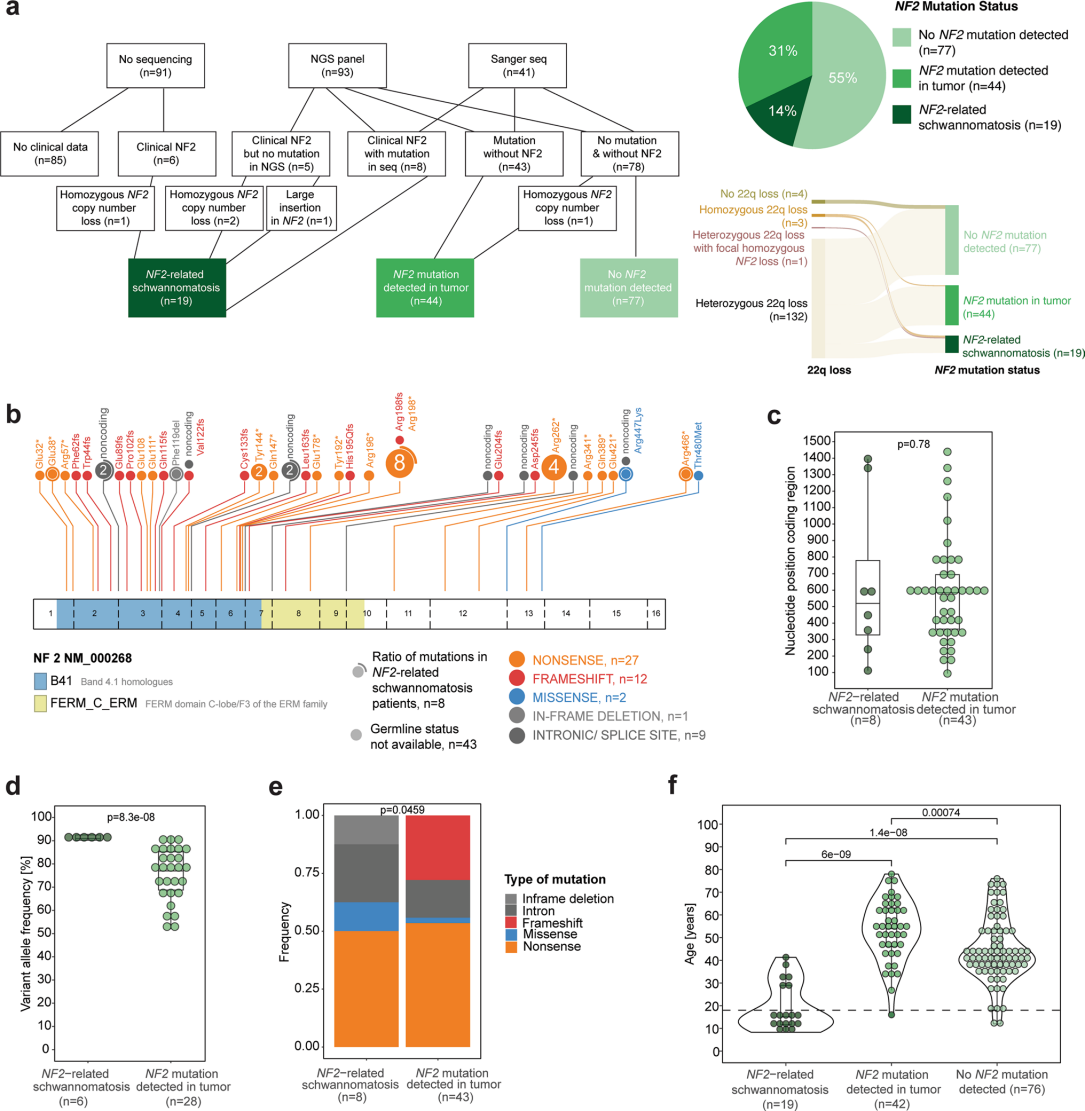
Landscape of NF2 mutations in SP-EPN. **a** Overview schematic of sequencing approaches and resulting mutational status of *NF2* (left) and pie chart representation of the detected mutational status of *NF2* (*n* = 140) based on sequencing of tumor material or clinical information (right top). Combined overview of 22q status together with detected mutational status of *NF2* visualized as Sankey plot (right bottom). **b** Lollipop plot depicting genomic distribution and type of germline and somatic *NF2* mutations in 51 spinal ependymoma as identified in NGS or Sanger sequencing. Dots with a circle outline represent mutations found in patients with *NF2*-related schwannomatosis, with the circumference indicating the ratio of germline mutations at this position. **c** Dot plot comparing the genomic position of *NF2* mutations between SP-EPN of patients with and without *NF2*-related schwannomatosis. Underlying boxplot displaying the first to the third quartile of the nucleotide position with a line in the middle representing the median value. **d** Dot plot comparing the variant allele frequencies of *NF2* mutations between SP-EPN of patients with and without *NF2*-related schwannomatosis. Underlying boxplot displaying the first to the third quartile of the variant allele frequency with a line in the middle representing the median value. **e** Bar plot representing the type of mutation between SP-EPN of patients with and without *NF2*-related schwannomatosis. **f** Violin plot comparing the age distribution of patients with *NF2*-related schwannomatosis, *NF2* mutations only found in the tumor or no *NF2* mutation detected in the tumor (*n* = 137). Underlying dot plot showing the age of each individual patient. Underlying boxplot displaying the first to the third quartile of the age distribution with a line in the middle representing the median value. Dotted line representing the cutoff for pediatric vs adult patients (18 years). Significance levels were determined using *t* test (**c**, **d**), Chi-square test (**e**) or Wilcoxon signed-rank test (**f**). *NF2 NF2*-related schwannomatosis

We next examined the genetic status of *NF2*, the only known driver of SP-EPN. Information on the *NF2* mutation status was available for 140 cases, either as sequencing result of tumor material (*n* = 134) or from clinical records about clinically or genetically confirmed NF2 (*n* = 6, Fig. 2a). We found underlying NF2 or mutations in *NF2* in 45% of cases (n = 63; Fig. 2a): 14% of SP-EPN samples were obtained from patients with known NF2 (*n* = 19) and 31% of samples had *NF2* mutations (*n* = 43) or homozygous loss of *NF2* (*n* = 1) that were detected only in tumor material with no available blood analysis and no documented clinical criteria of NF2 (Fig. 2a). In 55% (*n* = 77) of all analyzed samples, we detected no *NF2* mutation by sequencing (Fig. 2a). Copy number analysis revealed homozygous loss of chromosomal arm 22q in one case with no *NF2* mutation detected in sequencing (Suppl. Figure 1a). This case was considered “*NF2* mutation detected in tumor” for all following analyses. Additional Multiplex ligation-dependent probe amplification (MLPA) of 28 tumors without *NF2* mutation detected in sequencing did not detect any other homozygous chromosomal losses of *NF2* (data not shown). Of note, 5/19 SP-EPN samples that were obtained from patients with clinical *NF2*-related schwannomatosis did not show any *NF2* mutations in NGS panel analysis, suggesting that certain genomic alterations in *NF2* are not captured by NGS (Fig. 2a, left panel). Indeed, for two of these five samples, CNV analysis inferred from methylation data showed homozygous *NF2* loss (Suppl. Figure 1b–c). In one sample, a larger insertion of two exons of a foreign gene between exons 4 and 5 of *NF2* has been previously reported [25]. Importantly, since most SP-EPN cases showed heterozygous chromosomal 22q loss (Fig. 1h) in general, the majority of cases without detected *NF2* mutation represented tumors with loss of one *NF2* allele while the other allele was intact (*n* = 73/77,95%; Fig. 2a, right bottom panel). Only four cases showed neither *NF2* mutations nor chromosomal loss of *NF2* (Fig. 2a, right bottom panel). In contrast, all tumors with *NF2*-related schwannomatosis or *NF2* mutation detected in the tumor showed additional 22q loss, and thus bi-allelic loss of *NF2* (Fig. 2a, right bottom panel).

We next sought to characterize the spectrum of identified *NF2* mutations in SP-EPN (Suppl. Table 2). Of the 51 detected mutations, the majority (*n* = 39/51, 76%) were truncating mutations with 53% nonsense mutations (*n* = 27) and 24% frameshift mutations (*n* = 12) (Fig. 2b). The majority of *NF2* mutations occurred in the region encoding the plasma membrane binding FERM domain, spanning exon 1 to exon 10 of the *NF2* gene (*n* = 39/51, 76%), with mutation hotspots found in exon 6 at Arg198 as well as in exon 10 at Arg262 (Fig. 2b). The genomic distribution of mutations was similar for patients with *NF2* germline mutations and patients with mutations only detected in the tumor (Fig. 2c). Variant allele frequency (VAF) of *NF2* mutations ranged from 52 to 93% with significantly higher frequencies in tumors of patients with *NF2* compared to patients in whom the mutation was only found in the tumor (*p* = 8.3e–08; Fig. 2d). While the majority of *NF2* mutations were nonsense mutations in both groups, there were significant differences in the mutation types (*p* = 0.0459) with in-frame deletions only being detected in patients with *NF2*-related schwannomatosis (*n* = 1, 12.5%) and frameshift mutations only found in SP-EPN from patients without known NF2 (*n* = 12, 27%; Fig. 2e). When comparing clinical data across the different *NF2* groups, we found that age distribution differed significantly, with the lowest median age (15.1 years) in patients with *NF2*-related schwannomatosis and the highest median age (54.0 years) in patients with *NF2* mutations detected in the tumor (Fig. 2f). In contrast, anatomical location of SP-EPN did not show any differences between *NF2* groups (data not shown).

We next analyzed whether the spectrum of *NF2* mutations in SP-EPN differed from *NF2* mutations found in schwannoma and meningioma. We compiled published data of 102 schwannomas [2, 24] and 51 meningiomas [8, 17, 37], both comprising familial as well as sporadic cases. Of the mutation hotspots found in SP-EPN, the majority was affected in schwannomas and meningiomas, too (Suppl. Figure 2a, Suppl. Figure 3a). However, two genomic positions that were commonly altered in schwannomas [2, 24] were not found to be mutated in our SP-EPN data set: Tyr153/Asp154 and Leu361 (Suppl. Figure 2a). While *NF2* mutations in *NF2*-related schwannomatosis-related tumors showed similar genomic distribution in all three entities (SP-EPN, schwannoma and meningioma), mutations in sporadic schwannomas (or schwannoma without germline information) were located more towards the C-terminal end compared to SP-EPN with *NF2* mutations in tumor material (Suppl. Figure 2b, Suppl. Figure 3b). Although overall frequencies of mutation types mostly did not differ between the tumor entities, we found a significant difference between types of sporadic mutations (or mutations without germline information) with fewer frameshift mutations and more nonsense mutations detected in SP-EPN in comparison to schwannomas (Suppl. Figure 2c, Suppl. Figure 3c). Together, our genomic analysis reveals the frequency, genomic positions, and types of *NF2* mutations in SP-EPN in a large data set and identifies differences between *NF2* mutations in SP-EPN and other tumor entities.

### SP-EPN cells developmentally resemble mature ependymal cells

**Fig. 3:**
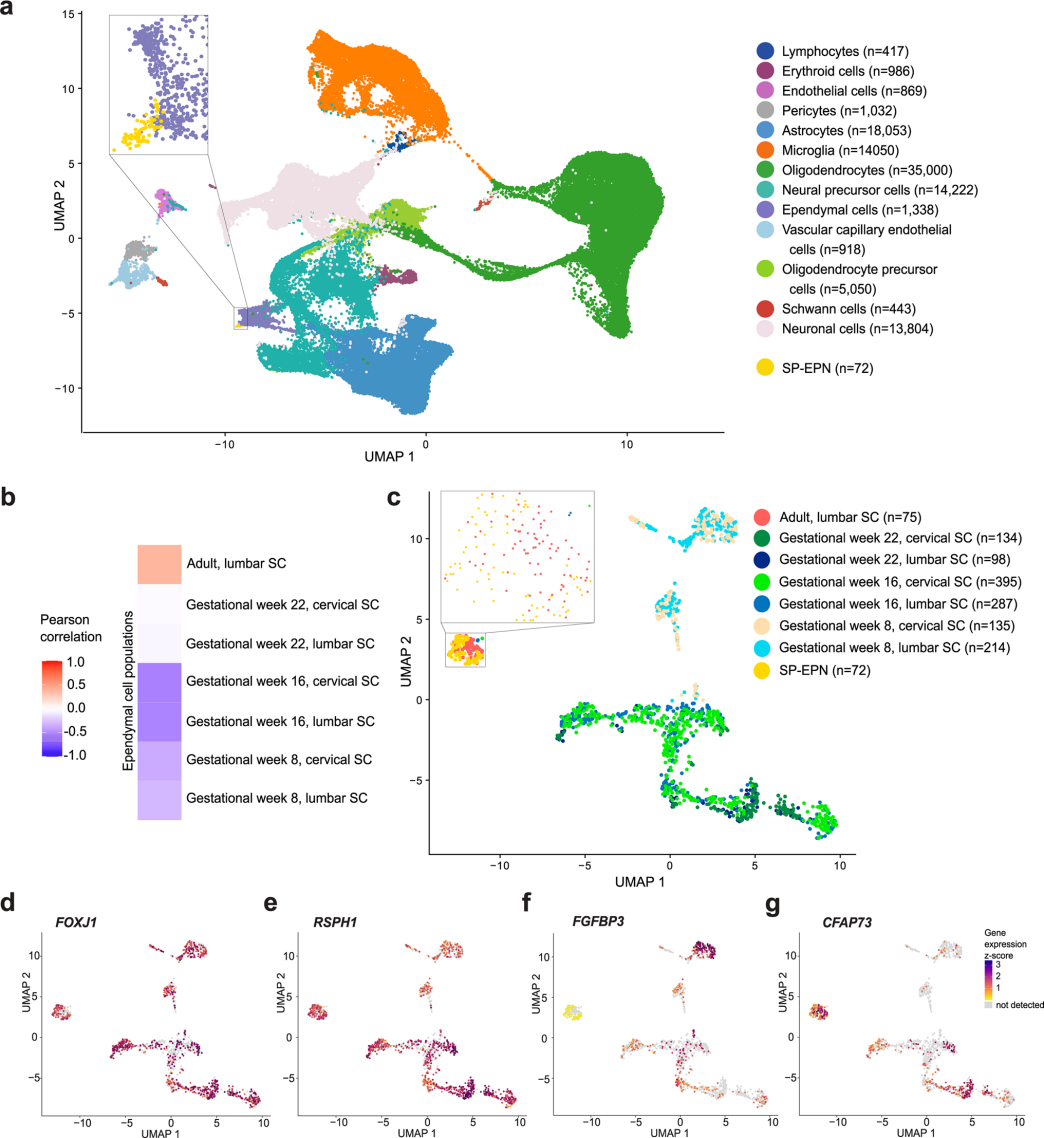
Developmental origin of SP-EPN. **a** UMAP projecting SP-EPN bulk RNA sequencing data into integrated single-cell and single-nucleus RNA sequencing reference data set. The reference includes human embryonal spinal cord of cervical and lumbar origin from gestational weeks 8, 16 and 22 (all *n* = 1) as well as human adult lumbar spinal cord (*n* = 7, age 50–80). Enlarged UMAP section on top left depicts localization of SP-EPN. **b** Pearson correlation analysis of SP-EPN samples with ependymal cell populations of different localization and age performed based on the 8000 most variable features. **c** Projection of SP-EPN bulk RNA sequencing data into UMAP of the ependymal cell cluster from (**a**). Enlarged UMAP section in upper left corner depicts localization of all SP-EPN. Expression heatmap of canonical ependymal marker genes *FOXJ1* **d**, *RSPH1* **e,** immature ependymal marker gene *FGFBP3* **f**, and adult ependymal marker gene *CFAP73* **g** of UMAP shown in (**c**). *SC* spinal cord

To gain insights into the cellular origin of SP-EPN for the first time, we compared the transcriptional profiles of 72 SP-EPN to an integrated reference data set of normal human spinal cord cell populations of various developmental stages. As a single-cell level reference data set, transcriptomic data from human embryonal spinal cord samples at gestational weeks 8, 16, and 22 were integrated with data from seven adult spinal cord samples [52, 58]. UMAP representation of the age- and localization-integrated reference together with bulk SP-EPN samples revealed that of all major cell types found in the spinal cord, ependymal cells showed closest transcriptional similarity to SP-EPN (Fig. 3a).

By performing Pearson correlation analysis and mapping tumors onto ependymal subpopulations, including different localizations and developmental stages, we analyzed the similarity between SP-EPN and ependymal cells in more detail (Fig. 3b–c). Among ependymal populations of different localization SP-EPN showed highest correlation with adult lumbar spinal cord (Fig. 3b). UMAP-based dimensionality reduction revealed that embryonal cells cluster based on age rather than anatomical localization (Fig. 3c). Additionally, SP-EPN again mapped closest to adult ependymal cells (Fig. 3c, enlarged section). SP-EPN showed high expression of canonical ependymal markers *FOXJ1* and *RSPH1* (Fig. 3d–e), generally verifying ependymal lineage of these tumors. The immature ependymal marker gene *FGFBP3* [29], which is not expressed in adult ependymal cells, showed only low expression levels in SP-EPN (Fig. 3f), whereas *CFAP73,* a marker for adult ependymal cells [52], was expressed in adult ependymal cells as well as in SP-EPN (Fig. 3g), again suggesting a mature ependymal transcriptional profile rather than earlier developmental origins of SP-EPN.

Overall, comparison of SP-EPN to spinal cord cell populations revealed transcriptional similarity of these tumors with adult mature ependymal cells.

### Transcriptomic profiling identifies two SP-EPN subtypes

**Fig. 4:**
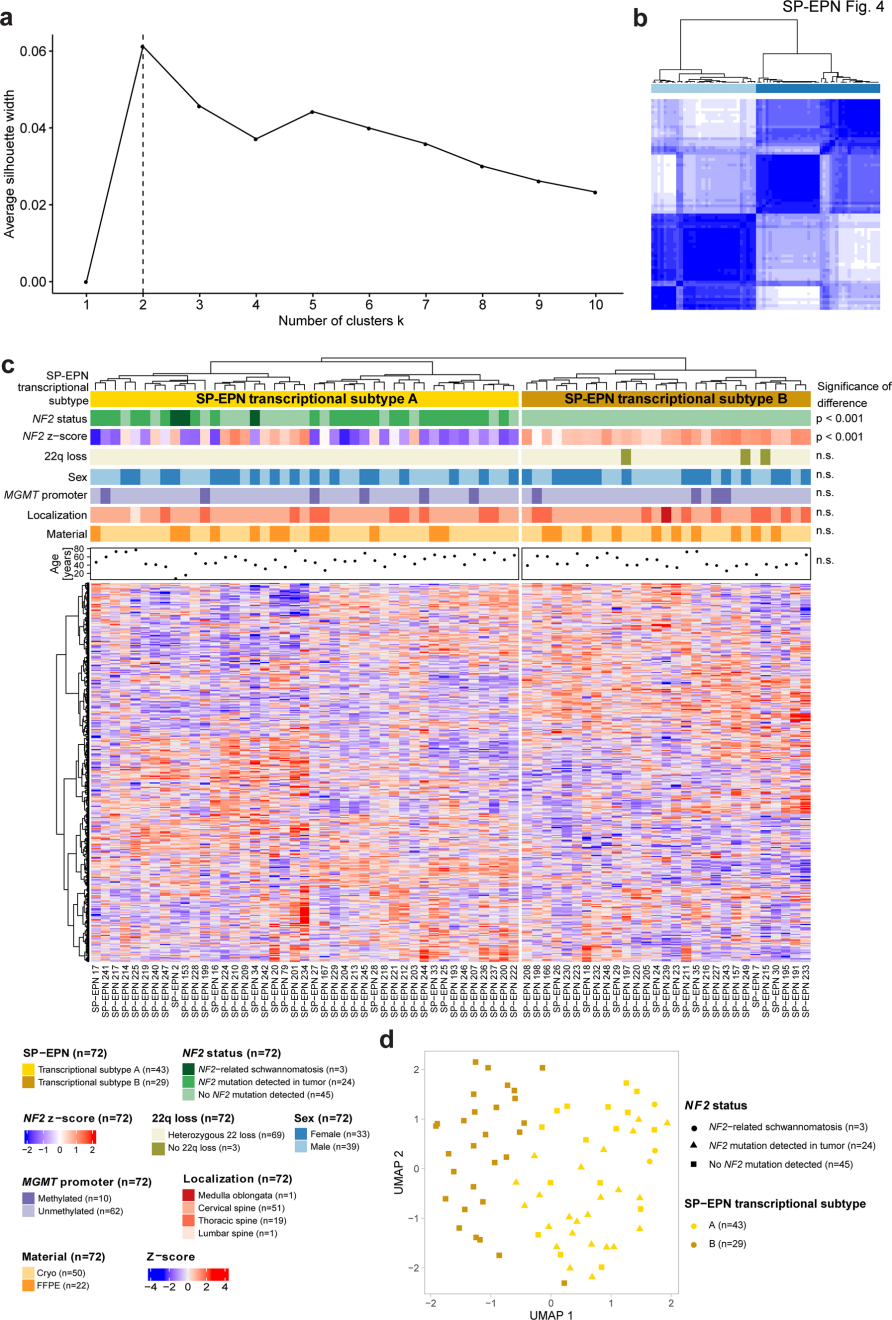
Identification of SP-EPN transcriptional subtypes. **a** Silhouette analysis of SP-EPN transcriptomic data (*n* = 72) determined the optimal cluster numbers of hierarchical clustering. **b** Consensus clustering matrix of SP-EPN transcriptomic data (*n* = 72). Heatmap displays cluster stability for samples that never cluster together (white) or always cluster together (blue) during hierarchical clustering of 1000 resamplings using 80% of tumor samples for each iteration. **c** Unsupervised hierarchical clustering of SP-EPN transcriptomic data (*n* = 72) based on the 4000 most variably expressed transcripts that are visualized as a heatmap of gene expression z-scores. Significance of difference was determined with Fisher's exact test for *NF2* status, 22q loss, *MGMT* promoter methylation, material type and tumor localization. Analysis of sex distribution was calculated with Pearson's Chi-squared test. Unpaired *t* test was used to evaluate differences in age and *NF2* expression *z* score. **d** UMAP showing the clustering of 72 SP-EPN using all detected transcripts (*n* = 19,271) for dimensionality reduction

To explore the intertumoral heterogeneity of SP-EPN, we further analyzed the transcriptome of SP-EPN. Clustering of bulk RNA sequencing data (*n* = 72, Suppl. Table 3) using the 4,000 most variable genes across the entire data set identified two transcriptional subtypes, as determined by silhouette analysis and consensus clustering (Fig. 4a–c). Clustering of cases was consistent between consensus clustering (Fig. 4b) and unsupervised hierarchical clustering (Fig. 4c) except for four cases (SP-EPN 203, 212, 221 and 244). These cases were part of cluster 1 of the consensus matrix, corresponding to transcriptional subtype B (Fig. 4b), but clustered together with transcriptional subtype cluster A in unsupervised hierarchical clustering (Fig. 4c).

*NF2* status differed significantly between the two transcriptional subtypes: SP-EPN subtype A comprised cases with *NF2*-related schwannomatosis, *NF2* mutations only in the tumor, and tumors with no *NF2* mutation detected, whereas SP-EPN subtype B only consisted of cases with no *NF2* mutation detected, (*p* < 0.001; Fig. 4c). Of note, SP-EPN subtype A contained only cases with heterozygous 22q loss (Fig. 4c, Suppl. Figure 4a), therefore presenting a mix of cases with bi-allelic loss of *NF2* (heterozygous 22q loss + *NF2* mutation) and cases with loss of only one *NF2* allele (heterozygous 22q loss + no mutation in *NF2* detected in sequencing). The majority of SP-EP subtype B tumors showed loss of one *NF2* allele (heterozygous 22q loss + no mutation in *NF2* detected in sequencing), while three SP-EPN subtype B cases had no 22q loss and no mutation in *NF2* detected in sequencing, indicating two intact *NF2* alleles (Fig. 4c, Suppl. Figure 4a).

Consistent with the difference in mutational and chromosomal status of *NF2* between the two SP-EP subtypes, expression of *NF2* was significantly lower in subtype A (*p* < 0.001; Fig. 4c, Suppl. Figure 4a). To support the presence of two distinct transcriptional subtypes, we performed UMAP-based dimensionality reduction, which confirmed the separation of subtype A and B (Fig. 4d).

To further characterize the two transcriptomic groups, differential gene expression analysis was performed (Suppl. Figure 4b, Suppl. Table 4). Of the 774 significantly differentially expressed genes identified between subtypes A and B, the most differentially expressed genes with higher expression in subtype A were *APOA1* (Log2FC = 3.85), which encodes a lipoprotein, *ADAMTS18* (Log2FC = 3.50), and *GREM1* (Log2FC = 3.38), both associated with brain development and differentiation [21, 61]*.* In addition, we found *FSTL3*, a known activator of Wnt /β-Catenin signaling, to be overexpressed in subtype A compared to subtype B tumors [30]. Interestingly, Wnt /β-Catenin signaling is a downstream effector in *NF2*-deficient tumors, too [60]. Genes with higher expression in subtype B were *NEUROD4* (Log2FC = − 5.03), encoding a known neuronal differentiation factor [45], *OR52E4* (Log2FC = − 4.48), an olfactory g-protein coupled receptor gene, and *SLC17A8* (Log2FC = − 4.45), which encodes a presynaptic glutamate transporter. As expected, *NF2* was among the differentially expressed genes with higher expression in SP-EPN subtype B (Suppl. Figure 4b).

Potential functional differences between groups were assessed by gene set enrichment analysis (GSEA) based on pathways annotated in the Reactome database [55] (Suppl. Figure 4c, Suppl. Table 5). For subtype A, “Regulation of Insulin-like Growth Factor (IGF) transport and uptake by Insulin-like Growth Factor Binding Proteins (IGFBPs)” and “Post-translational protein phosphorylation” showed highest enrichment (normalized enrichment score (NES) = 2.13 for both). Other significantly enriched terms in subtype A were associated with extracellular matrix, and nervous system development. Six pathway signatures were significantly enriched in subtype B, with the common themes related to the GO terms of “Sensory perception” and “Peptide-ligand binding receptors”. The highest NES was observed for the term “Class A/1 (Rhodopsin-like receptors)” (NES = -2.10). Of note, mapping and correlation of SP-EPN samples to spinal cord cell types of different localizations and developmental stages (Fig. 3) did not show any differences between the two SP-EPN subtypes, possibly indicating a similar cellular origin of both SP-EPN subtypes (data not shown).

To further verify our finding of two transcriptional SP-EPN groups in an independent data set, we used a publicly available set of transcriptomic microarray data comprising 209 ependymomas including 11 SP-EPN that were not included in our transcriptomic analysis (hereafter termed “DKFZ “ ependymomas, Suppl. Figure 4d) [36]. Hierarchical clustering was performed on all “DKFZ” ependymomas using the 774 significantly differentially expressed genes previously identified between our two SP-EPN subtypes (Suppl. Figure 4b). “DKFZ” ependymoma cases clustered according to molecular type, with SP-EPN splitting into two separate clusters. These two new clusters were assigned to the previously identified SP-EPN subtypes by comparing the expression profile of the two clusters to the top 10 differentially expressed genes for each SP-EPN subtype (annotated in Suppl. Figure 4b). Hence, the two identified transcriptional SP-EPN subtypes were verified using an external, independent cohort.

In addition to validating the newly identified SP-EPN subtypes, we identified genes with differential expression (i) between the two “DKFZ” SP-EPN clusters, corresponding to the two SP-EPN subtypes, as well as (ii) between one of the SP-EPN subtypes and other ependymoma types in the “DKFZ” data set (Suppl. Figure 4e–l). *BST1, FLG*, *KANK4,* and *SHISA9* were significantly higher expressed in SP-EPN subtype A compared to all other ependymomas, whereas *AK5*, *CFTR*, *RASSF6,* and *TBX2*2 showed high expression in subtype B.

Lastly, we compared immune cell abundance between the SP-EPN subtypes of our own data set based on transcriptomic signatures [7] (Suppl. Figure 5). Of the ten tested immune cell populations, significantly more T cells, CD8 T cells and natural killer (NK cells) were estimated for subtype B. Together, further analysis of the previously identified transcriptional SP-EPN profiles revealed variably enriched gene sets among the subtypes and validated the two subtypes in an independent cohort of 209 ependymoma cases.

### Two distinct molecular subtypes of SP-EPN can be validated by integrated analysis

**Fig. 5:**
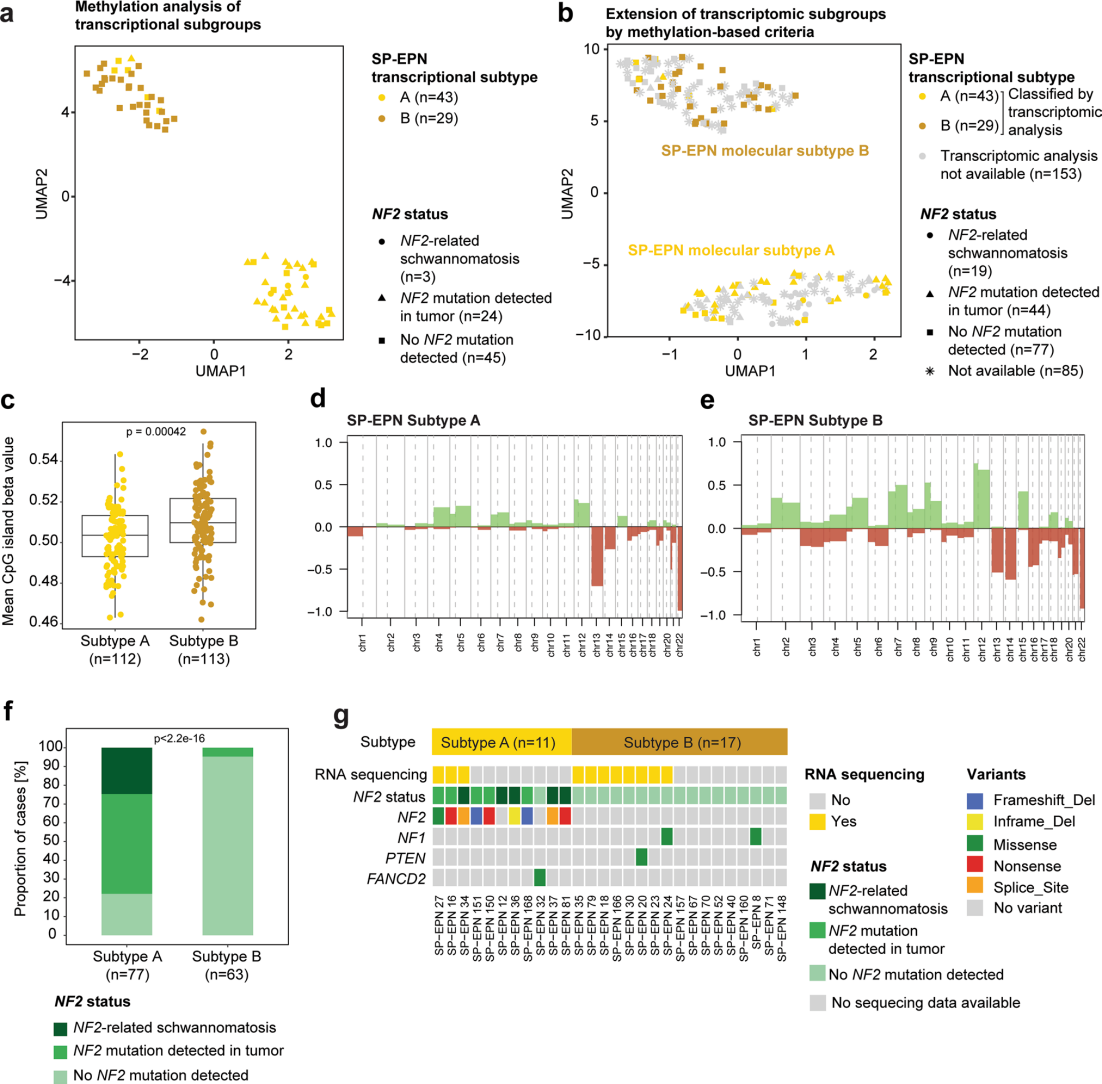
Integrated analysis of molecular SP-EPN subtypes. **a** UMAP displaying the clustering of methylation data based on 1518 differentially methylated CpG sites identified between the two transcriptional subtypes in SP-EPN with RNA sequencing data (*n* = 72). **b** UMAP of the complete SP-EPN cohort (*n* = 225) showing clustering based on 616 differentially methylated CpG sites identified between the two transcriptional subtypes. **c** Dot plot comparing mean methylation beta value based on global methylation of all CpG sites between the two molecular SP-EPN subtypes (*n* = 225). Underlying boxplot displaying the first to the third quartile of the mean methylation beta value of each sample with a line in the middle representing the median value for each SP-EPN subtype. **d** Overview of chromosome arm-wise copy number alterations in molecular subtype A (*n* = 112) and **e** subtype B (*n* = 113) of SP-EPN. **f** Bar plot showing *NF2* mutational status in subtype A (*n* = 77) and subtype B (*n* = 63) SP-EPN. **g** Oncoplot representing molecular subtype, *NF2* status and genomic variants classified as pathogenic/likely pathogenic (AMP Tier Class 2) of samples analyzed by WES (*n* = 28). Significance levels were determined using *t* test (**c**) or Fisher’s exact test (**f**)

To examine whether the two transcriptional subtypes harbor distinct molecular features that can be generally used to categorize SP-EPN, we integrated DNA methylation data, *NF2* sequencing, whole-exome sequencing, and histological analysis for 225 SP-EPN. Global DNA methylation profiling of the 72 SP-EPN cases that also had RNA sequencing data was used to identify 1,518 differentially methylated probes (DMP) that, with a few exceptions (*n* = 6/72, 8%), were able to robustly discriminate samples of transcriptional subtype A and B (Fig. 5a, Suppl. Table 6–7). UMAP dimensionality reduction of all 225 SP-EPN based on methylation of these DMP confirmed two molecular subtypes in the methylation data set (Fig. 5b), reflecting the previously discovered transcriptional differences between the two subtypes (Fig. 4a–c). For further molecular and clinical analyses of the entire cohort, we therefore defined extended molecular subtypes SP-EPN A and B based on methylation changes that were found between the two transcriptional subtypes (Fig. 5b). Since the methylation-based subtypes showed a clear overlap with the previously identified transcriptional subtypes (*n* = 66/72, 92%; Fig. 5b; Suppl. Table 7), we continued with the same nomenclature (SP-EPN molecular subtype A and B). To analyze global methylation in an unbiased way, we performed UMAP dimensionality reduction based on the 10,000 generally most variable CpG sites which confirmed methylation-based discrimination of the two transcriptional subtypes in the subset of SP-EPN with RNA sequencing and in the extended SP-EPN cohort (Suppl. Figure 6a-b). However, the separation was not as clear as with the integrated approach based on DMP previously identified in a supervised manner (Fig. 5a–b). To further validate the two molecular SP-EPN subtypes (Fig. 5b), we analyzed additional molecular characteristics based on methylation analysis. Global methylation level, defined as mean methylation of all CpG island beta values, was significantly higher in SP-EPN subtype B (Fig. 5c). Interestingly, arm-wise copy number alterations were more common in SP-EPN subtype B, presenting with a median of 14 alterations per sample, whereas SP-EPN subtype A tumors harbored a median of only six copy number alterations per sample (Fig. 5d–e). Both groups shared a high incidence of losses of chromosome 22q (Fig. 5d–e, Suppl. Figure 6c–d), followed by losses of chromosome 13 and 14 (Fig. 5d–e). In addition, SP-EPN subtype B more commonly showed gains of chromosome 2, 5, 7, 8, 9, 12, and 15, as well as loss of chromosome 16 compared to subtype A tumors (Fig. 5d–e). *MGMT* promoter methylation, histological diagnosis, anatomical location did not differ between SP-EPN subtypes (Suppl. Figure 6e–g). Even though age generally did not differ between the subtypes, the majority of tumors in pediatric patients were SP-EPN subtype A tumors (*n* = 19/21, 90%; Suppl. Figure 6h).

We next sought to examine the genomic landscape of the two methylation-based SP-EPN subtypes. Subtype A comprised mostly of tumors with bi-allelic *NF2* loss (60/77, 78%) – these cases had either underlying *NF2*-related schwannomatosis or *NF2* mutations detected in the tumor, all harboring chromosomal loss of 22q. (Fig. 5f, Suppl. Figure 6c). Only a small fraction of subtype A cases (17/77, 22%) harbored monoallelic *NF2* loss, as they presented without *NF2* mutation together with 22q loss (Fig. 5f, Suppl. Figure 6c). In contrast, Subtype B consisted mostly of tumors with loss of one *NF2* allele (heterozygous 22q loss with no *NF2* mutation detected) (Fig. 5f, Suppl. Figure 6d). Only three SP-EPN subtype B cases showed bi-allelic *NF2* loss and (22q loss with *NF2* mutation in tumor) and four cases showed intact *NF2* status (no 22q loss and no *NF2* mutation detected in sequencing) (Fig. 5f, Suppl. Figure 6d). Thus, comparison of genomic and chromosomal *NF2* status between SP-EPN molecular subtype A and B largely confirmed the previous findings of the transcriptional subtypes (Fig. 4c–d). To identify additional genomic alterations, we performed whole-exome sequencing (WES) of 28 SP-EPN covering both molecular subtypes (SP-EPN subtype A: *n* = 11, SP-EPN subtype B: *n* = 17) as well as different *NF2* statuses. Notably, apart from the previously known *NF2* mutations, other likely pathogenic genomic alterations (classified as AMP Tier class 1 or 2) in known oncogenes or tumor suppressor genes were extremely rare, with only two additional variants found in *NF1* (p.V1432I, p.P1400L), and one variant found in *PTEN* (p.L230W) and *FANCD2* (p.R794*) each (Fig. 5g). Neither mutations in the tumor suppressor gene *PTEN,* which is commonly altered in glioma [46], nor in *FANCD2*, which is essentially involved in DNA repair, have been described in ependymomas yet [4, 13]. In addition to these variants, we detected a higher number of variants of unknown significance (VUS). We analyzed gene ontology (GO) enrichment for these variants and performed clustering of GO terms based on semantic similarities. The biologically most relevant, broadest GO term for each cluster was then manually selected to compare enriched GO terms between the two SP-EPN subtypes. We found that both subtypes had VUS in genes associated with cytoskeleton organization, cell adhesion, gliogenesis, sodium ion transport, and extracellular matrix organization (Suppl. Figure 6i–j, Suppl. Table 8). Subtype A tumors had additional VUS in genes implicated in cell cycle, cell division and smoothened signaling pathway, whereas subtype B tumors additionally showed VUS in genes involved in transmembrane receptor protein serine/threonine kinase signaling pathway and synapse organization (Suppl. Figure 6i–j, Suppl. Table 8).

To assess whether the distinct molecular characteristics also manifest in morphological differences between the two SP-EPN subtypes, we examined histopathological features of 43 SP-EPN in a blinded manner. While tumors generally presented with heterogeneous morphological characteristics (Suppl. Figure 7a–k), molecular subtype A more commonly exhibited pleomorphic cell nuclei, whereas subtype B tumors more frequently showed ependymal surfaces, high cell density, and fielded growth (Suppl. Figure 7 l).

Overall, integrated analysis of SP-EPN combining DNA methylation profiling, DNA sequencing, and histological assessment confirmed the presence of two subtypes with distinct molecular and histopathological features.

### Molecular SP-EPN subtypes show different clinical outcomes and therapeutic vulnerabilities

**Fig. 6:**
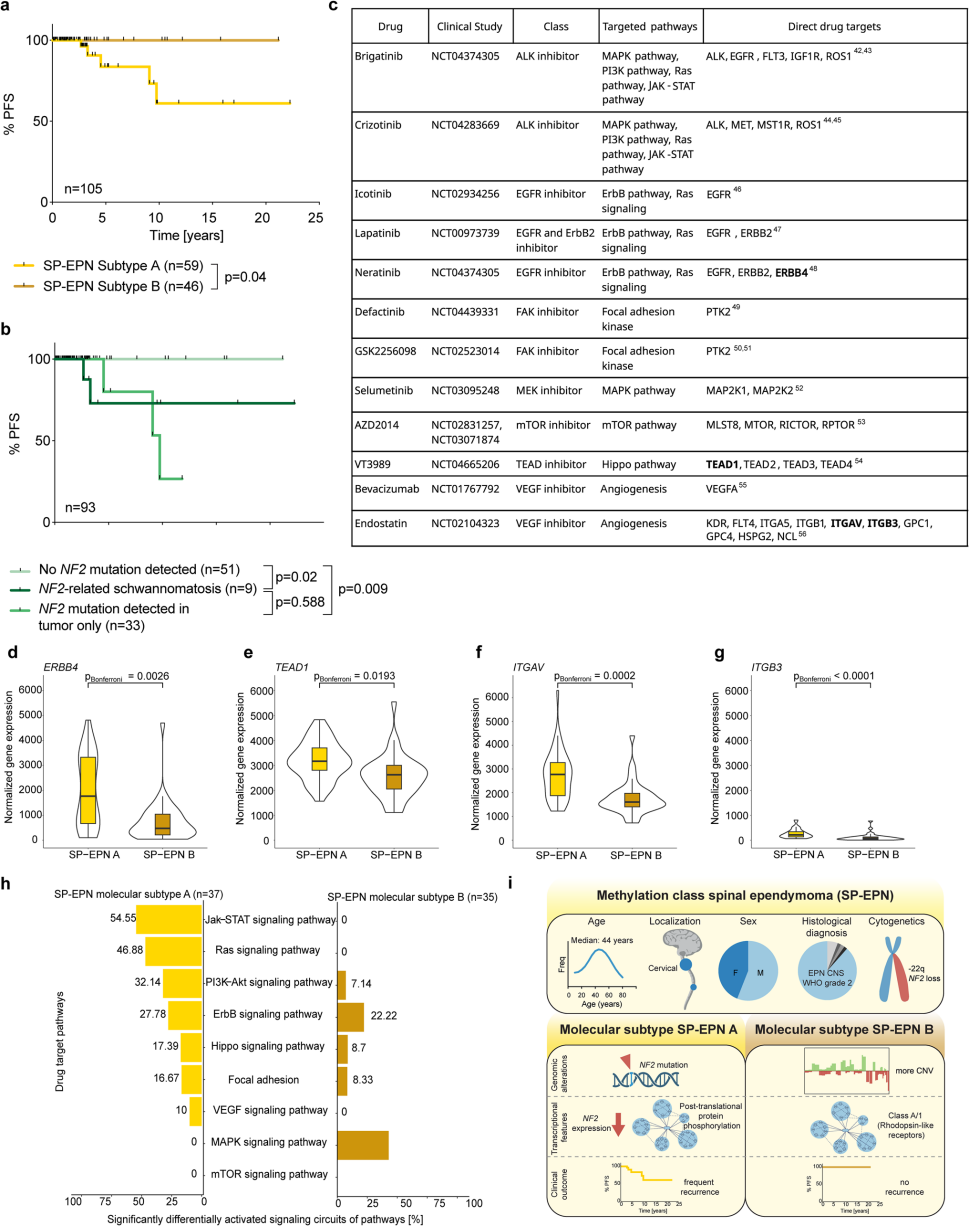
Clinical relevance of SP-EPN subtypes. **a** Kaplan–Meier curve displaying progression-free survival (PFS) of patients with molecular subtype A and B SP-EPN (*n* = 59 and *n* = 46, respectively). Significance was calculated by Log-rank test. **b** Kaplan–Meier curve showing PFS of patients with no *NF2* mutation detected (*n* = 51), *NF2* mutation detected in the tumor (*n* = 33), or *NF2*-related schwannomatosis (*n* = 9). *P* values were calculated by Log-rank test. **c** Table depicting drugs that are currently in clinical trials for *NF2*-associated tumors together with their inhibitor type, targeted pathways and direct drug targets. Violin Plots of DEseq2-normalized gene expression of significantly differentially expressed drug target genes *ERBB4* (**d**), *TEAD1* (**e**), *ITGAV* (**f**) and *ITGB3* (**g**). Underlying boxplot displaying the first to the third quartile of the normalized gene expression of each sample with a line in the middle representing the median value for each SP-EPN subtype. Significance between molecular subtypes A (*n* = 37) and B (*n* = 35) was tested with unpaired Wilcoxon test and adjusted for multiple testing applying the Bonferroni method. **h** Canonical circuit activity analysis for drug target pathways identifying the fraction of differentially activated signaling circuits of whole pathways between SP-EPN molecular subtypes. **i** Overview on the demographic and pathological features of SP-EPN in general as well as on the two SP-EPN molecular subtypes with their different molecular and clinical characteristics. Displayed GO-terms in the “transcriptional features” section are the top enriched GO-terms in each SP-EPN subtype

Clinical outcome of SP-EPN is generally considered favorable, but retrospective analyses of larger cohorts are lacking. Therefore, we assessed the clinical outcome of 112 epigenetically defined SP-EPN cases and further correlated the data with the molecular subtype (Suppl. Table 9). Median follow-up was 25 months, ranging from 1 to 267 months. Overall survival (*n* = 112) for all SP-EPN was excellent and did not differ between molecular subtypes (Suppl. Figure 8a). Of the two patients who deceased in this cohort, one patient died from complications of a tetraparesis, most likely caused by the progression of his SP-EPN. The other patient, a patient with NF2, was diagnosed with multiple other tumors including a highly aggressive malignant peripheral nerve sheath tumor (MPNST), which presumably caused his death. When examining progression-free survival (PFS, *n* = 105), we found a significant difference between SP-EPN of the two molecular subtypes: Tumor progression or relapse occurred only in SP-EPN molecular subtype A, whereas all subtype B patients remained progression-free (*p* = 0.04, Fig. 6a). Since subtype B predominantly comprises tumors with no *NF2* mutation detected, we next compared the outcome of patients according to the *NF2* status of their tumors (*n* = 93). Notably, PFS differed significantly between patients with tumors harboring *NF2* mutations that were only detected in the tumor and patients with SP-EPN with no *NF2* mutation detected (*p* = 0.009; Fig. 6b) as well as between cases with no *NF2* mutation detected and *NF2*-related schwannomatosis patients (*p* = 0.02; Fig. 6b). Within SP-EPN subtype A, PFS did not significantly differ between the distinct *NF2* groups, potentially due to smaller case numbers (*n* = 51, Suppl. Figure 8b).

When analyzing additional clinical parameters, we found that SP-EPN subtype A tumors showed a trend towards more cases with only subtotal resection and more cases that required post-operative radiation, although both were not significant (Suppl. Figure 8c–d). In addition, they more often occurred as multiple tumors as compared to SP-EP subtype B (Suppl. Figure 8e), overall suggesting a clinically more aggressive phenotype of SP-EPN subtype A tumors.

Although our data confirm a generally good survival of SP-EPN, we found that the majority of patients not only presented with initial symptoms including paralysis of the lower limbs or urinary incontinence (Suppl. Figure 8f) but also commonly developed new postoperative complications such as neurological impairment (Suppl. Figure 8g), emphasizing the high clinical burden and negative impact on quality of life. Therefore, there is an urgent need to establish treatment approaches that can help alone or in combination with surgery to reduce tumor growth and alleviate symptoms in patients with SP-EPN.

To identify potential therapeutic vulnerabilities in the two molecular SP-EPN subtypes, we queried clinical trial databases and literature to select compounds that were currently or recently being investigated in clinical trials for ependymomas or *NF2*-related schwannomatosis-associated tumors (Fig. 6c). We next compared expression of known direct target genes (*n* = 31, listed in Fig. 6c) of these compounds [3, 12, 18, 20, 23, 32, 40, 43, 44, 48, 50, 53, 54, 56, 59] and found four target genes to be significantly differentially expressed between the two molecular subtypes, indicating a potentially different therapy response to the respective drugs. Genes *ERBB4* (targeted by EGFR inhibitor Neratinib), *TEAD1* (targeted by TEAD inhibitor VT3989), *ITGAV,* and *ITGB3* (targeted by endogenous anti-angiogenic inhibitor Endostatin) showed higher expression in SP-EPN subtype A (Fig. 6d-g). None of the direct drug target genes was significantly higher expressed in SP-EPN B. Next, we assessed differential activation of signaling circuits within the pathways targeted by the drugs mentioned above (Fig. 6c) in SP-EPN subtype A and B (Fig. 6h). For most pathways, SP-EPN subtype A showed more differentially activated signaling circuits (JAK-Stat, Ras, PI3K-Akt, ErbB, Hippo, Focal adhesion and VEGF), indicating that drugs targeting these pathways might be of particular benefit in these clinically more aggressive tumors (Fig. 6h). However, the activity of signaling circuits of the MAPK pathway was significantly lower in subtype A tumors, suggesting that drugs targeting the MAPK pathway might not be as efficient in these cases (Fig. 6h).

In summary, we identified differences in clinical outcome and other clinical parameters between the molecular SP-EPN subtypes. Furthermore, we revealed that the expression of several target genes of compounds tested in clinical trials and the activity of pathways targeted by these drugs differ between the two groups, indicating potential clinical utility of the newly identified molecular subtypes. The molecular as well as demographic characteristics of our SP-EPN cohort and the two newly identified SP-EPN subtypes are synoptically shown in Fig. 6i.

## Discussion

The discovery and characterization of distinct molecular intracranial and spinal ependymoma types and subtypes have expanded our understanding of ependymoma pathobiology and may contribute to refining diagnosis and risk stratification of ependymoma patients. Here, we profiled a large SP-EPN cohort using genomic, transcriptomic, and epigenetic approaches that were integrated with histopathological and clinical data. Overall, our data not only illuminates intertumoral heterogeneity and developmental origins of SP-EPN, but also, importantly, reveals, for the first time, a subset of tumors with worse clinical outcome (SP-EP subtype A).

The only known drivers of SP-EPN are mutations in the tumor suppressor gene *NF2* in combination with chromosomal loss of 22q, resulting in bi-allelic loss of *NF2*, as per Knudson’s two-hit hypothesis [5, 13, 51]. Most data on the prevalence of *NF2* mutations and 22q loss in spinal ependymal tumors were generated prior to the identification of distinct molecular ependymoma types and therefore show varying frequencies of these driver events [5, 13, 28, 42]. In our cohort, we observe *NF2* mutations or clinical *NF2*-related schwannomatosis in 45% of all SP-EPN, while 96% of all tumors show loss of 22q. The high frequency of 22q loss suggests pathogenic relevance even without a second “hit”, potentially through reduced *NF2* gene dosage. To further test this hypothesis, *NF2* expression of SP-EPN cases with monoallelic loss (22q loss without additional *NF2* mutation) could be compared to expression levels in normal ependymal cells with intact genomic *NF2* status in future studies.

Clinical data or prior germline testing indicated underlying NF2 in 14% of all cases. However, we cannot entirely rule out that among the cases with *NF2* mutations detected in tumor material, there were more cases with NF2, as complete clinical records were not available for all patients and germline testing was not performed. Since no *NF2* mutation was detected in 55% of SP-EPN (monoallelic *NF2* loss), our data suggest that other drivers might contribute to the pathogenesis of these tumors. While neither NGS nor WES revealed recurrent mutations in other tumor suppressor or oncogenes in our data set, we cannot rule out that larger WES studies might identify rare mutations in SP-EPN. However, we frequently found copy number alterations, especially in SP-EPN subtype B which mainly consisted of tumors without *NF2* mutation (monoallelic *NF2* loss), possibly contributing to tumorigenesis of these cases. The majority of *NF2* mutations in SP-EPN were N-terminal and truncating variants (76% each). In *NF2*-related schwannomatosis, these two features—truncating mutation and N-terminal position—are associated with a clinically more severe phenotype with younger age at disease presentation and more frequent peripheral nerve tumors, spinal tumors and meningiomas (Whishart phenotype) [19]. However, this phenotype-genotype correlation has not yet been shown for sporadic *NF2* mutations.

In addition to providing a thorough characterization of the genomic landscape of SP-EPN, this study shows developmental similarities of these tumors to mature adult ependymal cells for the first time, suggesting their developmental origin in rather late stages of ependymal differentiation. A recent single-cell transcriptomic study demonstrated that in two other low-grade ependymoma types with favorable outcome, MPE and posterior fossa-B (PFB) ependymoma, differentiated ependymal-like cells were the most frequent malignant cell type [16]. In contrast, more aggressive supratentorial and posterior fossa ependymoma types contained higher proportions of more immature glial-progenitor-like and neuronal-precursor-like cells in the same study [16]. Therefore, our findings showing high similarities between SP-EPN and mature adult ependymal cells further support the hypothesis that less aggressive ependymoma types, such as SP-EPN, consist of more differentiated ependymal-like cells whereas aggressive ependymomas are mainly composed of more immature progenitor-like cells [16]. Mapping the transcriptomes of malignant cells to single-cell atlases of distinct cell populations during CNS development could not only offer novel information about the developmental origin of these tumors but could also provide insights into new therapeutic strategies aiming at cell-intrinsic mechanisms specific to this developmental stage.

The identification of distinct transcriptional and methylation-based subtypes has transformed the definition of almost every CNS tumor entity, such as glioblastoma, medulloblastoma, and ATRT [22, 33, 46]. Here, we identified two novel molecular subtypes within SP-EPN that were robustly distinguishable using several clustering approaches of transcriptomic data. These two SP-EPN subtypes strongly correlated with mutation status and expression of *NF2*, indicating that distinct global transcriptional profiles might reflect different genomic driver events of SP-EPN. Furthermore, integrated analysis revealed differential methylation of a subset of CpG sites between the two transcriptional subtypes, which was then used to classify an extended data set of SP-EPN. In this way, we confirmed the presence of two molecular SP-EPN subtypes based on methylation analysis that largely overlapped with the two transcriptional subtypes. Specifically, *NF2* genomic status significantly differed between the two molecular subtypes, too, with subtype A harboring mostly cases with bi-allelic *NF2* loss (germline or sporadic mutations in *NF2* together with 22q loss) and subtype B comprising predominantly cases with monoallelic *NF2* loss (no *NF2* mutation detected in addition to 22q loss). This was reflected in differences in *NF2* expression, too, with subtype A tumors showing decreased expression levels, which could potentially serve as a biomarker for distinguishing the two subtypes. However, when analyzing global methylation in an unbiased way, separation of the two transcriptional groups was less clear, suggesting that SP-EPN are epigenetically more homogenous while showing clear transcriptional differences. In addition, the two novel subtypes exhibited distinct clinical outcomes. SP-EPN subtype A tumors, which histologically displayed a higher frequency of pleomorphic nuclei, more often showed progression or recurrence, more often required post-operative radiation therapy and more often occurred as multilocular disease, indicating a clinically more aggressive phenotype of SP-EPN subtype A. Hence, while patients with SP-EPN subtype B display excellent progression-free survival after surgical resection, this current standard of care treatment might not be sufficient for all patients with SP-EPN subtype A. Lastly, we analyzed pathway activity and expression of genes targeted by small molecule inhibitors that have already been tested in clinical studies for *NF2*-related tumors or ependymomas and found significant differences between the two SP-EPN subtypes. Therefore, knowledge of the underlying molecular characteristics of SP-EPN might inform more specific clinical use of these inhibitors in the future.

### Supplementary Information

Below is the link to the electronic supplementary material.

Supplementary file1 (PDF 6250 KB)

Supplementary file2 (XLSX 9549 KB)

Supplementary file3 (PDF 100 KB)

## Data Availability

Raw and processed data are available under GEO GSE242994.
