# Supplementary material for: Transcriptomic and epigenetic dissection of spinal ependymoma (SP-EPN) identifies clinically relevant subtypes enriched for tumors with and without NF2 mutation
Source: Acta Neuropathol. 2024 Jan 24;147(1):22. doi: 10.1007/s00401-023-02668-9 (PMC10808175; doi:10.1007/s00401-023-02668-9)
Supplement: Supplementary file 1 — Supplementary file1 (PDF 6250 KB) [file 401_2023_2668_MOESM1_ESM.pdf]

Transcriptomic and epigenetic dissection of spinal ependymoma (SP-EPN) identifies clinically relevant subtypes enriched for tumors with and without *NF2* mutation

Acta Neuropathologica

Sina Neyazi, Erika Yamazawa, Karoline Hack et al.

Corresponding authors: Ulrich Schüller (u.schueller@uke .de), Department of Pediatric Hematology and Oncology, University Medical Center Hamburg-Eppendorf, Germany; Shota Tanaka (stanaka@m.u-tokyo.ac.jp), Department of Neurosurgery, Graduate School of Medicine, The University of Tokyo, Japan

Supplemental figures

SP-EPN Suppl Fig 1

a

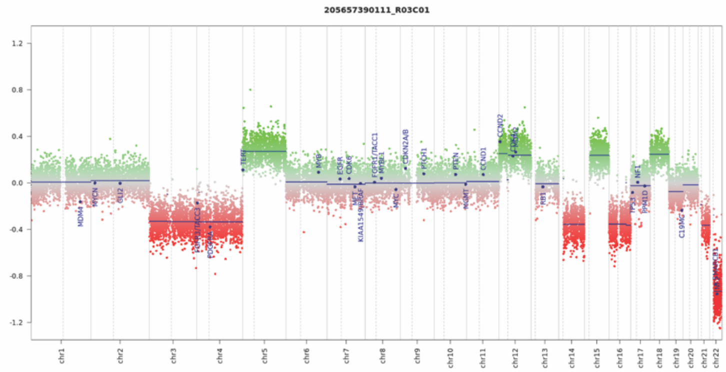

b

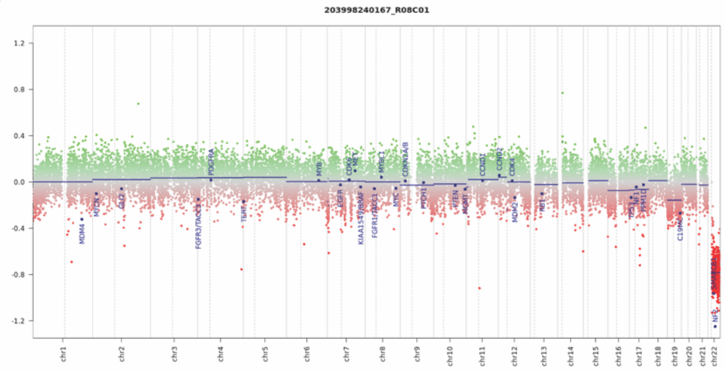

c

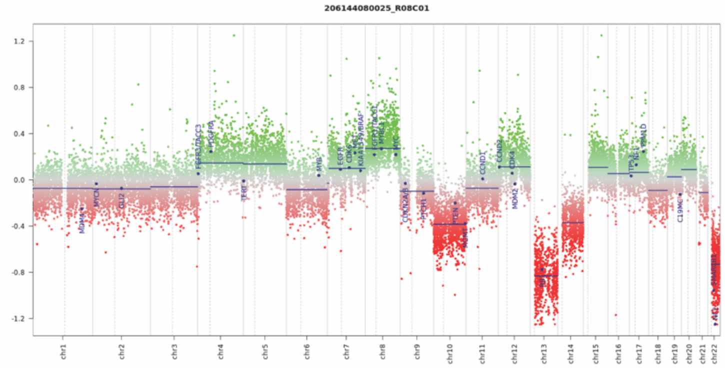

d

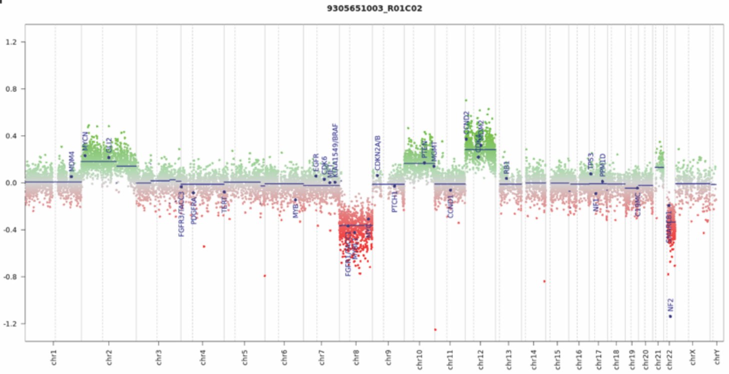

SP-EPN Suppl Fig 2

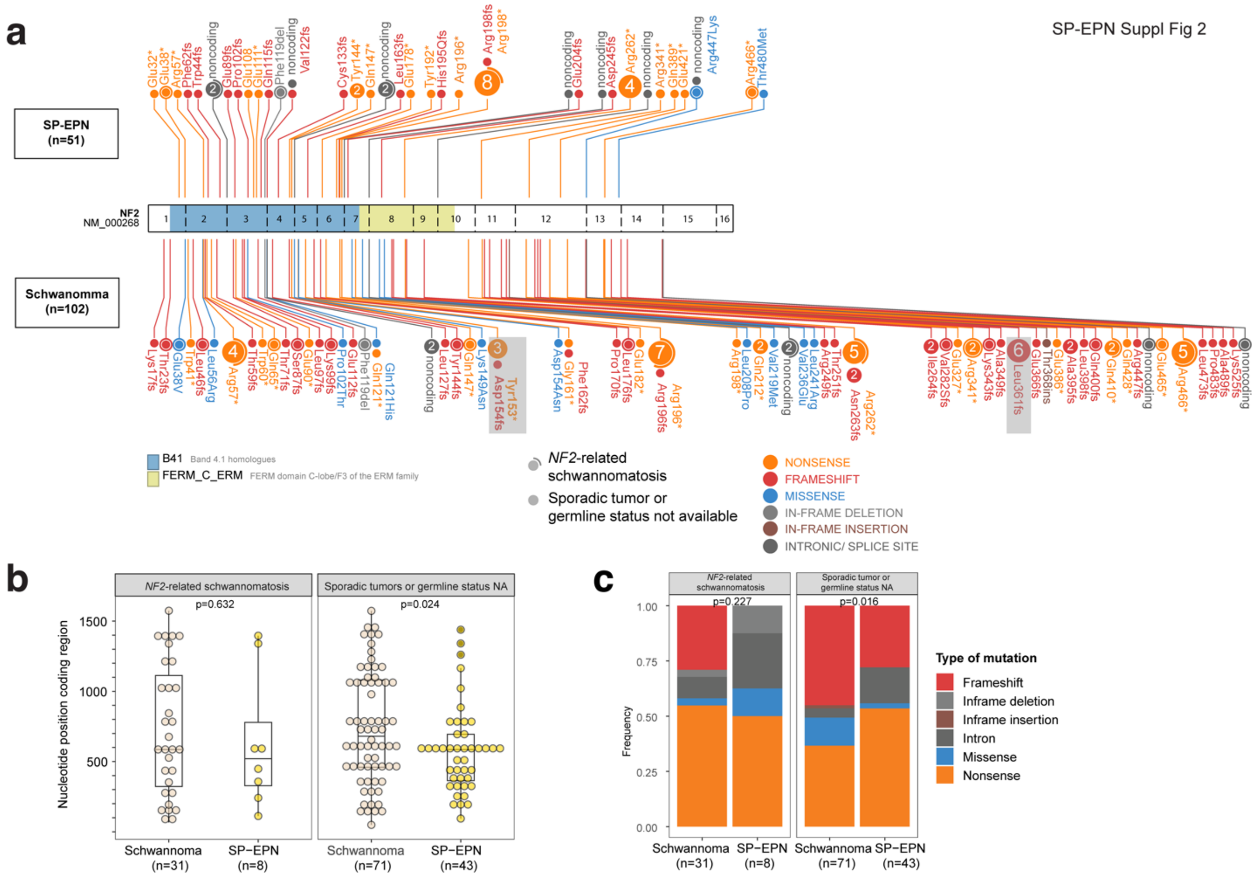

SP-EPN Suppl Fig 3

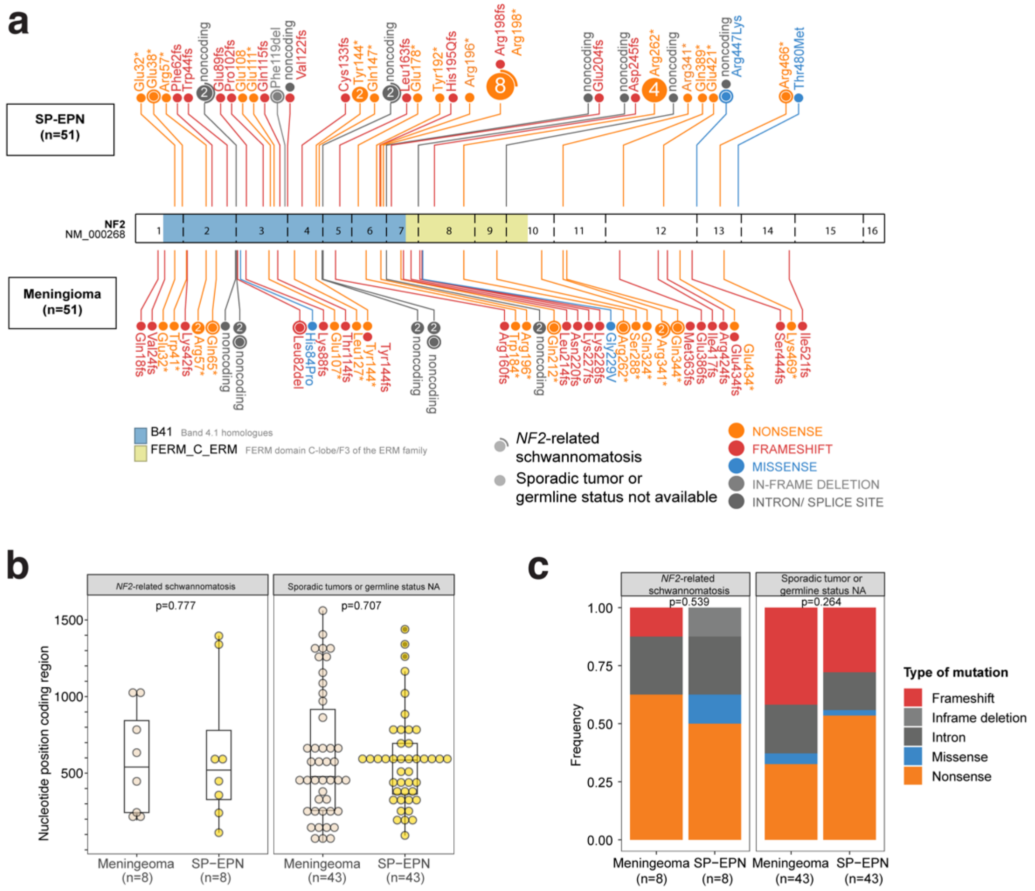

SP-EPN Suppl Fig. 4

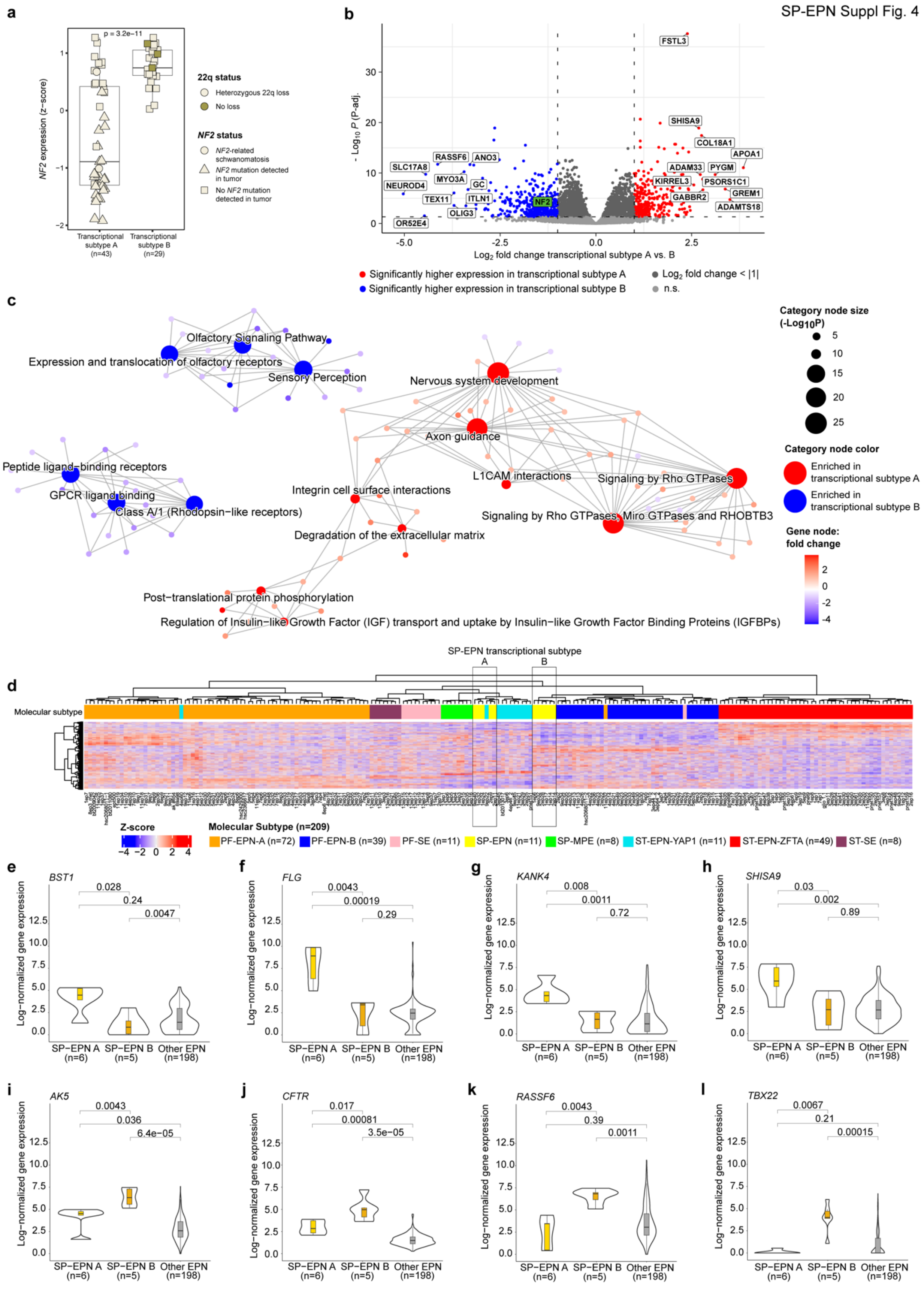

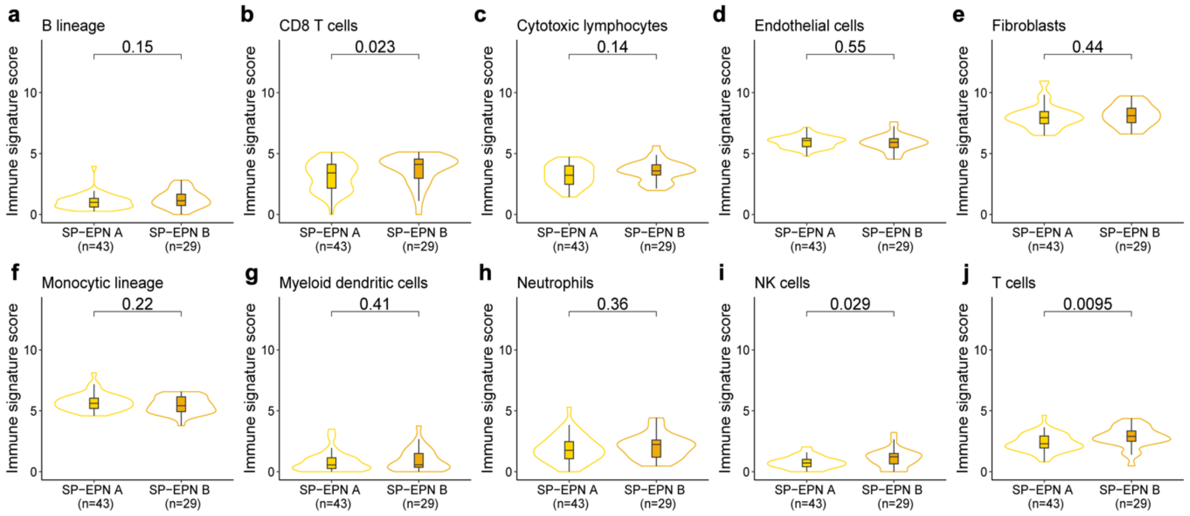
a
B lineage
0.15
b
CD8 T cells
0.023
c
Cytotoxic lymphocytes
0.14
d
Endothelial cells
0.55
e
Fibroblasts
0.44
f
Monocytic lineage
0.22
g
Myeloid dendritic cells
0.41
h
Neutrophils
0.36
i
NK cells
0.029
j
T cells
0.0095
Immune signature score
0
5
10
SP−EPN A (n=43)
SP−EPN B (n=29)

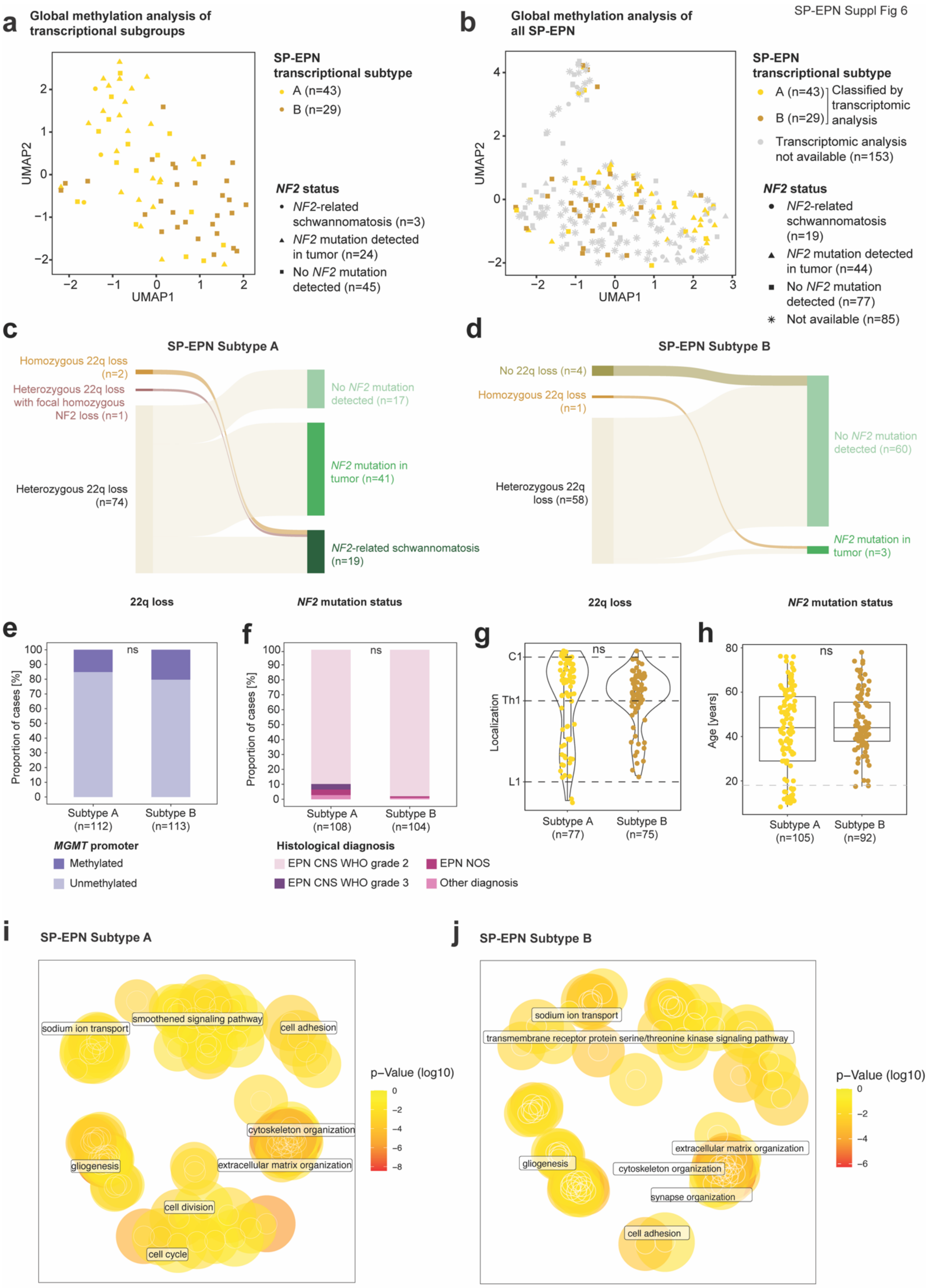
a
Global methylation analysis of transcriptional subgroups
UMAP2
UMAP1
SP-EPN transcriptional subtype
A (n=43)
B (n=29)
NF2 status
NF2-related schwannomatosis (n=3)
NF2 mutation detected in tumor (n=24)
No NF2 mutation detected (n=45)
b
Global methylation analysis of all SP-EPN
SP-EPN transcriptional subtype
A (n=43)
B (n=29)
Classified by transcriptomic analysis
Transcriptomic analysis not available (n=153)
NF2 status
NF2-related schwannomatosis (n=19)
NF2 mutation detected in tumor (n=44)
No NF2 mutation detected (n=77)
Not available (n=85)
c
SP-EPN Subtype A
Homozygous 22q loss (n=2)
Heterozygous 22q loss with focal homozygous NF2 loss (n=1)
Heterozygous 22q loss (n=74)
No NF2 mutation detected (n=17)
NF2 mutation in tumor (n=41)
NF2-related schwannomatosis (n=19)
22q loss
NF2 mutation status
d
SP-EPN Subtype B
No 22q loss (n=4)
Homozygous 22q loss (n=1)
Heterozygous 22q loss (n=58)
No NF2 mutation detected (n=60)
NF2 mutation in tumor (n=3)
22q loss
NF2 mutation status
e
Proportion of cases [%]
ns
Subtype A (n=112)
Subtype B (n=113)
MGMT promoter
Methylated
Unmethylated
f
Proportion of cases [%]
ns
Subtype A (n=108)
Subtype B (n=104)
Histological diagnosis
EPN CNS WHO grade 2
EPN CNS WHO grade 3
EPN NOS
Other diagnosis
g
Localization
C1
Th1
L1
ns
Subtype A (n=77)
Subtype B (n=75)
h
Age [years]
ns
Subtype A (n=105)
Subtype B (n=92)
i
SP-EPN Subtype A
sodium ion transport
smoothened signaling pathway
cell adhesion
cytoskeleton organization
gliogenesis
extracellular matrix organization
cell division
cell cycle
p−Value (log10)
j
SP-EPN Subtype B
sodium ion transport
transmembrane receptor protein serine/threonine kinase signaling pathway
extracellular matrix organization
gliogenesis
cytoskeleton organization
synapse organization
cell adhesion
p−Value (log10)

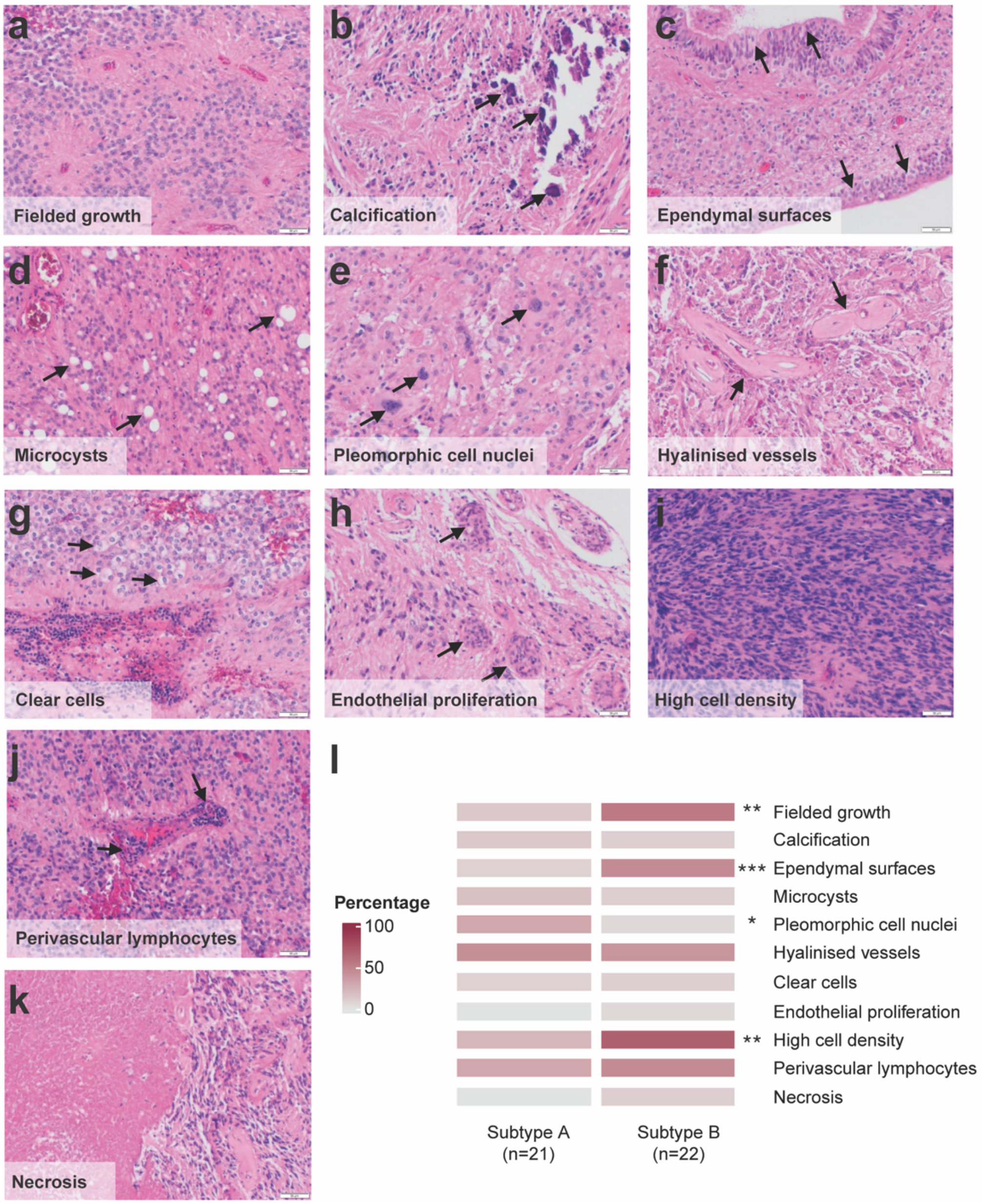
a
Fielded growth
b
Calcification
c
Ependymal surfaces
d
Microcysts
e
Pleomorphic cell nuclei
f
Hyalinised vessels
g
Clear cells
h
Endothelial proliferation
i
High cell density
j
Perivascular lymphocytes
k
Necrosis
l
Percentage
100
50
0
** Fielded growth
Calcification
*** Ependymal surfaces
Microcysts
* Pleomorphic cell nuclei
Hyalinised vessels
Clear cells
Endothelial proliferation
** High cell density
Perivascular lymphocytes
Necrosis
Subtype A
(n=21)
Subtype B
(n=22)

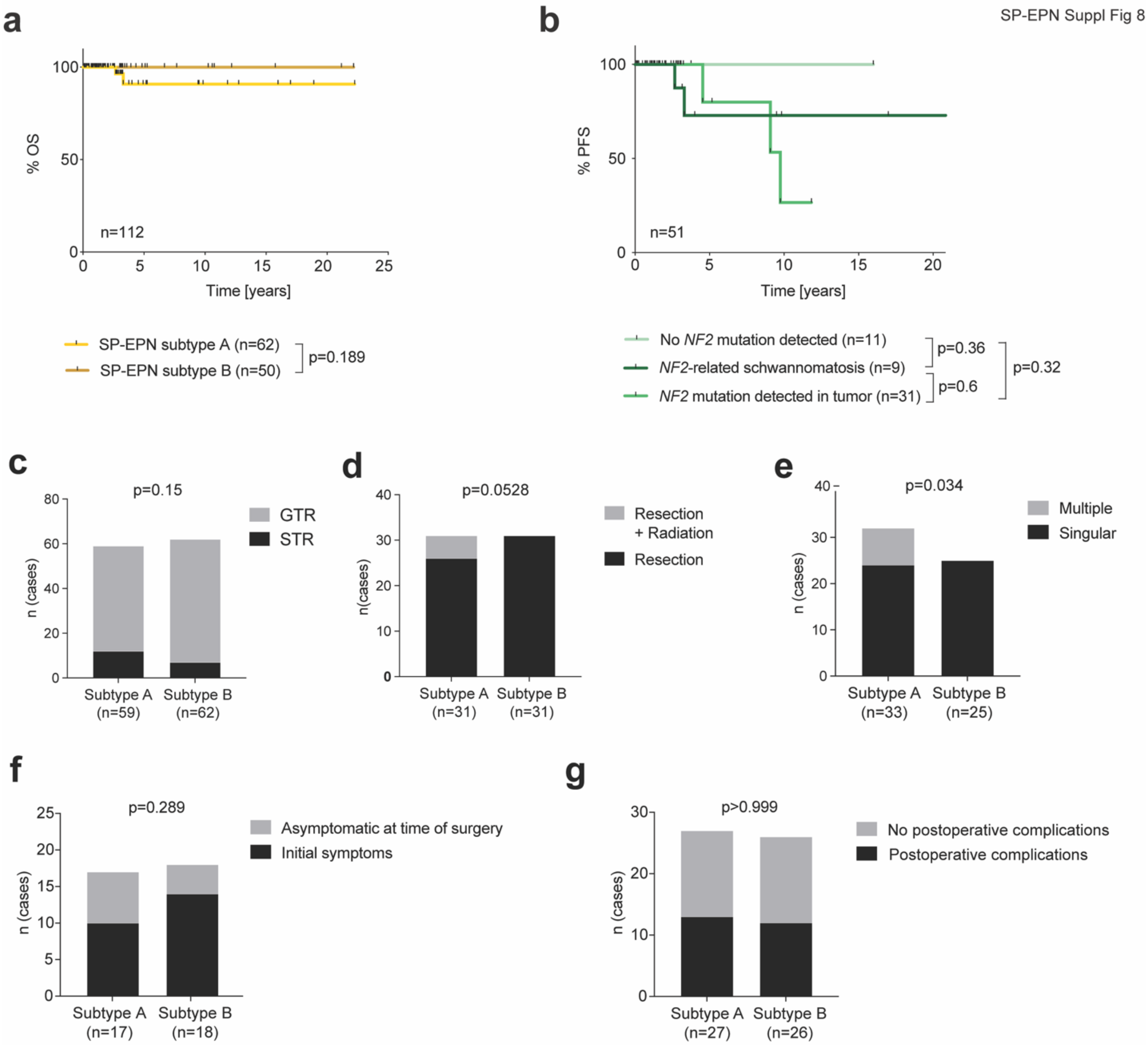

a
100
50
0
% OS
n=112
0 5 10 15 20 25
Time [years]
SP-EPN subtype A (n=62)
SP-EPN subtype B (n=50)
p=0.189
b
100
50
0
% PFS
n=51
0 5 10 15 20
Time [years]
No NF2 mutation detected (n=11)
NF2-related schwannomatosis (n=9)
NF2 mutation detected in tumor (n=31)
p=0.36
p=0.6
p=0.32
c
p=0.15
n (cases)
GTR
STR
Subtype A (n=59)
Subtype B (n=62)
d
p=0.0528
n(cases)
Resection + Radiation
Resection
Subtype A (n=31)
Subtype B (n=31)
e
p=0.034
n (cases)
Multiple
Singular
Subtype A (n=33)
Subtype B (n=25)
f
p=0.289
n (cases)
Asymptomatic at time of surgery
Initial symptoms
Subtype A (n=17)
Subtype B (n=18)
g
p>0.999
n (cases)
No postoperative complications
Postoperative complications
Subtype A (n=27)
Subtype B (n=26)
