# Supplementary material for: Transcriptomic and epigenetic dissection of spinal ependymoma (SP-EPN) identifies clinically relevant subtypes enriched for tumors with and without NF2 mutation
Source: Acta Neuropathol. 2024 Jan 24;147(1):22. doi: 10.1007/s00401-023-02668-9 (PMC10808175; doi:10.1007/s00401-023-02668-9)
Supplement: Supplementary file 3 — Supplementary file3 (PDF 100 KB) [file 401_2023_2668_MOESM3_ESM.pdf]

Transcriptomic and epigenetic dissection of spinal ependymoma (SP-EPN) identifies clinically relevant subtypes enriched for tumors with and without *NF2* mutation

Acta Neuropathologica

Sina Neyazi, Erika Yamazawa, Karoline Hack et al.

Corresponding authors: Ulrich Schüller (u.schueller@uke .de), Department of Pediatric Hematology and Oncology, University Medical Center Hamburg-Eppendorf, Germany; Shota Tanaka (stanaka@m.u-tokyo.ac.jp), Department of Neurosurgery, Graduate School of Medicine, The University of Tokyo, Japan

<u>Supplemental methods</u>

Genome-wide DNA methylation profiling and data processing

DNA was extracted from FFPE tissue using the ReliaPrep™ FFPE gDNA Miniprep System (Promega). About 100-500 ng DNA were used for bisulfite conversion by the EZ DNA Methylation Kit (Zymo Research). The DNA Clean & Concentrator-5 (Zymo Research) and the Infinium HD FFPE DNA Restore Kit (Illumina) were employed to clean and restore the converted DNA. The methylation status of 450,000 or 850,000 CpG sites was analyzed on the iScan system using the HumanMethylation450 BeadChip array or the Infinium MethylationEPIC BeadChip Kit (both Illumina), respectively. If DNA methylation profiling had been performed locally or elsewhere, raw data were made available for this study.

Methylation data were processed using minfi and noob normalization. CpG sites associated with SNPs, sex chromosomes, cross-reactive sites, and sites that were not represented on the 450k or the EPIC BeadChip were excluded as described previously[1]. Previously published samples that were present multiple times in the cohort, were identified based on their SNP profile using *getSnpBeta* in minfi and subsequently excluded. The predicted match to the methylation class “SP-EPN” was obtained using the “Heidelberg” brain tumor classifier developed by Capper et al[1] (https://www.molecularneuropathology.org/mnp/). Classifier version [v12.5] was used and the cut-off value of ≥0.9 was implemented to indicate a significant match. Samples with detection p-values ≥0.01 were excluded from the cohort.

For unsupervised clustering of SP-EPN cases with other EPN cases, as well as for unfiltered clustering of methylation data of all SP-EPN cases, the 10,000 most variable CpG sites were used. Differentially methylated probes (DMP) between the two SP-EPN RNA subtypes were determined with ChAMP[2], using adjusted (Benjamini-Hochberg procedure for multiple comparisons) p-values <0.05 and logFC <-0.2 or >0.2 as thresholds. 1,518 DMP were identified,

of which 616 were also covered by the Infinium HumanMethylation450 BeadChip that was used for metyhylation analysis of a subset of SP-EPN samples. All SP-EPN cases were subsequently clustered based on the methylation of these 616 DMP between the two RNA subtypes to establish extended molecular subtypes. UMAPs were generated with *umap* using the default parameters. Copy number plots were generated using *Conumee* and *GISTIC2* for the segmentation of arm-level methylation-based copy number profiles with default parameters[3,4]. One case was excluded for copy number analysis because of low quality in the copy number plot. A threshold of -0.1 was applied to detect relevant losses of chromosomal arm 22q. For all cases with 22q values between 0 and -0.2, manual assessment of CNV plots was additionally performed to validate 22q copy number status. The threshold for homozygous loss of 22q and *NF2* was -0.75. *MGMT* promoter methylation status was assessed as described previously[1].

Sanger sequencing

PCR was performed using KAPA Taq Extra kit (Kapa Biosystems) and confirmed by gel electrophoresis (1% agarose gel at 100V for 15 min). PCR products were purified by Illustra ExoProStar Kit (Cytiva) and further processed with BigDye™ Terminator v3.1 Cycle Sequencing Kit (Applied Biosystems). Reactants were then purified using BigDye XTerminator™ Purification Kit (Applied Biosystems) and incubated at 2000 rpm for 30 min. Sequencing was performed at the Center for Genomic Medicine, The University of Tokyo Hospital. DNADynamo software (Blue Tractor Software) was used to analyze the results.

Primers for Sanger Sequencing:

NF2 exon 1	Forward primer	GGGCTAAAGGGCTCAGAGTG
	Reverse primer	ACCTCTCGAGCTTCCACCTC
NF2 exon 2	Forward primer	AGTGTCATCCCCACGTTTTG
	Reverse primer	CCCCAGTGATGAGCTAGGC
NF2 exon 3	Forward primer	TTGCAAAGGCTTCTTTGAGG
	Reverse primer	AACTCTGCAACCACTCCTGG
NF2 exon 4	Forward primer	GCCATCTGTTGTGATCAGCC
	Reverse primer	TCCCATGACCCAAATTAACG
NF2 exon 5	Forward primer	TGTTCAGAAATGGCAGTTATCTTTAG
	Reverse primer	CCTTCAAGTCCTTTGGTTAGC
NF2 exon 6	Forward primer	CTCTGTGTGACTATCTCCCTGG

	Reverse primer	CAAGCATGTCCTAGTTTTGCAG
NF2 exon 7	Forward primer	AATGCTTGATTTGGTGCCC
	Reverse primer	AGTCTGGCCCTCACTCAGTC
NF2 exon 8	Forward primer	GTGCCAGATTCTTTGGAAGG
	Reverse primer	GGGGCAGACAGGGAAAG
NF2 exon 9	Forward primer	CCAATTGCTGGTAACATTCC
	Reverse primer	CATTCTATACTTCACAAGATGTCACTC
NF2 exon 10	Forward primer	TAGTGGGCCAGTAGGCAGTG
	Reverse primer	GGCCAGGACTGACCACAC
NF2 exon 11	Forward primer	CTTGTGGCACCCTAGGTCTC
	Reverse primer	TCAAAGCAAACGCTGCTAAC
NF2 exon 12	Forward primer	CCCATCTCAGTGTTCAAGGC
	Reverse primer	CTGGTCTGCGGCCCTTC
NF2 exon 13	Forward primer	TGTCCTTTTTCACCTCTTTGG
	Reverse primer	GTGTTTGCCTGAATGGTCAC
NF2 exon 14	Forward primer	AGGATCGGTTGTCAACACAG
	Reverse primer	AGGCCCCAATCACTCAGTC
NF2 exon 15	Forward primer	GAGCCGTGTCTCACTGTCTG
	Reverse primer	AGGAAACCAGATGCCAACC
NF2 exon 16	Forward primer	AGGACAGGACCCTGTGTGAC
	Reverse primer	TTGATATCTGGTCCATCCCG

DNA panel-based sequencing and data processing

Next-generation DNA sequencing was performed using a customized targeted gene panel, manufactured by Qiagen (CDHS-21330Z-424). The panel covers the complete coding regions and splice-sites of six genes (*ATRX, EGFR, NF1, NF2, PTEN, TP53*), as well as mutation hotspots of further 14 genes (*AKT, BRAF, CTNNB1, FGFR1, FGFR2, H3F3A, HIST1H3B, HIST1H3C, IDH1, IDH2, KRAS, PI3CA, PIK3R1, TERT* promoter). Library preparation was done according to the manufacturer's instructions. Samples were sequenced on the Illumina MiniSeq sequencing system (paired-end, 2 x 151 bp, average coverage 500x). Data were analyzed with the Qiagen CLC Genomics workbench, using a customized workflow. Variants were annotated with information from the 1000 genome project, dbSNP, ClinVar and COSMIC. Only variants with an allele frequency ≥5% and a total target coverage of ≥40x were further analyzed. Variants not

annotated by ClinVar were additionally assessed with VarSome (www.varsome.com). Lollipop-Plots were created using the web application on https://proteinpaint.stjude.org/.

Whole-exome sequencing and data processing

DNA was isolated from FFPE tissues using the ReliaPrep™ FFPE gDNA Miniprep System (Promega). 50 ng DNA were used for library preparation (Human Core Exome Plus Kit, Human RefSeq Panel, and Mitochondrial Panel; all Twist Bioscience). Samples were sequenced on the Nova Seq 6000 System (Illumina) (paired-end, 2x 100 bp). Demultiplexing of sequenced reads was performed using *bcl2fastq* (Illumina) and adapter trimming using *Skewer*. Trimmed raw reads were aligned to reference genome hg19-cegat using *Burrows-Wheeler-Aligner*. Local realignment of reads in target areas was performed with *ABRA* to achieve more accurate indel calling. Proprietary software (cegat) was used to discard duplicate reads, which originate most likely from the same PCR amplicon, and reads that were aligned with the same mapping score at more than one location in the genome. For variant calling, another proprietary software (cegat) was used. Identified variants were annotated based on publicly available data bases. Variants were further filtered for variants with an annotated population frequency <0.0001 and an allele frequency ≥ 0.3. Variants occurring in introns, UTRs or classified as synonymous variant were excluded. Putative clinical relevance of detected, filtered variants was determined based on annotation in ClinVar and Varsome. Variants classified as “benign” or “likely benign” were further excluded. Gene ontology analysis of genes affected by the remaining variants was performed using the *DAVID* functional annotation tool with the following thresholds: count=2, ease = 0.05. Enriched gene ontology terms were clustered and visualized with *Revigo*[5].

Bulk RNA sequencing

RNA of SP-EPN was isolated from FFPE tissues using the Maxwell® RSC RNA FFPE Kit (Promega). Concentrations were measured with a Qubit 2.0 Fluorometer (Thermo Fisher Scientific) and RNA integrity was analyzed using the RNA 6000 Nano Kit on a 2100 Bioanalyzer (Agilent Technologies).

After ribosomal RNA depletion and library preparation (SMART-Seq Stranded Kit, Takara Bio), samples were sequenced on the Nova Seq 6000 System (Illumina) (paired-end, 2x 100 bp).

Pre-processing was started by checking RNA sequencing read quality using FASTQC (v0.11.9) and subsequently aligned to the human reference genome assembly (GRCh38.106) using STAR aligner (v2.7.10a) in default 1-pass mode. Mapped reads were quantified using featureCounts

(v1.6.0) as part of the Subread software package. The “isPairedEnd” parameter was specified in default “unstranded” read counting mode.
Affymetrix HG U133 Plus 2.0 microarray data of 209 ependymoma[7] were downloaded from GEO (GSE64415) as MAS5.0 normalized and log2 transformed counts.

Bulk RNA sequencing data processing

All analyses were performed in R. Summarized read counts (SP-EPN n=61,552; microarray EPN data n = 54,675 transcripts total) with transcript IDs were mapped to HUGO Gene Nomenclature Committee (HGNC) symbols using *BiomaRt*. Non-protein coding transcripts and those without HGNC annotation were removed. The means of transcripts that matched the same HGNC symbol were calculated and genes of the Y chromosome were excluded to avoid gender bias. 19,271 transcripts for SP-EPN, and 16,779 for microarray EPN data were retrieved.
For SP-EPN samples, batch effects were detected based on principal component analysis (PCA) and subsequent correction was performed between the five different hospitals, where the samples were collected. The *ComBat_seq* function of the *sva* package was used on raw count data to remove batch effects. Afterwards, batch corrected counts were normalized using the “median of ratios” method of the *DESeq2* package[8]. For clustering analyses, a subsequent variance stabilization transformation followed by calculation of z-scores was performed. To obtain the 4,000 genes for clustering of SP-EPN, the most variable genes within each batch were determined first to reduce the influence of technical variability between batches on the analysis. Therefore, variance stabilization transformation was performed on each batch individually and 3,000 genes with highest intra-batch variance were determined and the union taken. For each gene, the variance within each batch was weighted by batch size and for this purpose multiplied by sample size per batch/ total sample size. Afterwards, we calculated the sum for each gene of the weighted variance of all batches. Genes were ordered by the resulting batch-weighted variance and the 4,000 genes with the highest values were selected for clustering analysis.
The average Silhouette width was determined by the *factoextra::fviz_nbclust* function and for consensus clustering the *ConsensusClusterPlus* package was employed[9]. Here, hierarchical clustering using the options “ward.D2” and “pearson” was performed with 1000 resamplings, each on 80% of samples and all features.
ComplexHeatmap visualized hierarchical clustering of SP-EPN using Ward’s method (D2) and Pearson distance[10,11]. UMAP dimensionality reduction was performed on all features and variance stabilized counts (19257 transcripts) with *n_neighbors* set to 15 using *umap*.

For differential expression analysis between SP-EPN subtype A and B, *DESeq2::DESeq* was used on batch-corrected counts. The volcano plot of differentially expressed genes was created with *EnhancedVolcano*[12]. Significantly differentially expressed genes were defined by adjusted p-Value < 0.05 and log2 fold change (log2FC) >1.

Reactome pathway gene set enrichment analysis (GSEA)[13] was conducted on the significantly differentially expressed genes ranked by log2FC using *ReactomePA::gsePathway*[14] . Significantly enriched pathways were selected with Benjamini-Hochberg adjusted p-value <0.1. The gene network plot was compiled by *enrichplot::cnetplot*.

For hierarchical clustering of microarray data of 209 EPN tumors, the count matrix was subset to differentially expressed genes from comparison of SP-EPN subtypes A and B (n=774) and z-scores were calculated. *ComplexHeatmap* with Ward's method (D2) for clustering based on Pearson distance was applied on tumor samples. The two resulting SP-EPN clusters were matched to subtypes by evaluation of the expression pattern of the 20 most differentially expressed genes between SP-EPN subtypes (Top 10 genes for SP-EPN subtype A: APOA1, ADAMTS18, GREM1, PYGM, GABBR2, PSORS1C1, COL18A1, ADAM33, SHISA9, KIRREL3; Top 10 genes for SP-EPN subtype B: NEUROD4, OR52E4, SLC17A8, RASSF6, TEX11, OLIG3, MYO3A, ITLN1, GC, ANO3).

To determine potential marker genes of each SP-EPN cluster, differential expression was calculated between SP-EPN cluster tumors and all other ependymomas from the analysis using *limma*. Significantly differentially expressed genes were selected with Benjamini-Hochberg adjusted p-value <0.01 and logFC >1. Afterwards, only genes that also demonstrated significant differential expression in the comparison of transcriptional subtypes A and B in our cohort of 72 bulk RNA sequencing SP-EPN were considered as potential marker genes for subtypes.

Immune cell abundance in SP-EPN samples was estimated as described by Bockmayr et al.[15] The DESeq2-normalized and log2 transformed counts of SP-EPN were used for calculation of immune scores. Differential pathway activation analysis was conducted utilizing *HiPathia*[16], which performs a canonical circuit activity analysis. Variance stabilized count data of SP-EPN were scaled to values between 0 and 1 with *hipathia::normalize_data.* When calculating signaling circuit activation, only effector pathways were included and differential activation was tested with *hipathia::do_wilcoxon.*

Single-cell/single-nucleus RNA sequencing analysis

Human adult lumbar spinal cord single nucleus RNA sequencing data of seven donors[17] were downloaded from GEO repository GSE190442 as aggregated counts. Human embryonal cervical

and lumbar spinal cord single cell RNA sequencing samples[18] were selected from GEO repository GSE136719 (GSM4080448, GSM4080410, GSM408043, GSM4080449, GSM4080411, GSM4080437). All reference samples were integrated using the standard *Seurat* v4 workflow[19]. Quality control was performed by selecting cells with nFeatures >200 and <3000 and excluding cells with more than 5% mitochondrial transcripts. Default parameters were used for integration of the first 30 principal components of the reference samples and *seurat_clusters* were determined at a resolution of 0.5. Differences between G2/M and S cell cycle phases were removed as recommend by Seurat vignette “Cell-Cycle Scoring and Regression”, where cell cycle scores are calculated based on specific marker genes[20].

As Yadav et al. provided a thorough annotation, these data were employed in the process of cell type annotation of the embryonal data. Furthermore, cluster gene expression was compared to appropriate literature[17,18,21].

DESeq2-normalized bulk SP-EPN RNA sequencing data were subjected to Seurat v4 reference-based mapping workflow[19,22], where the SP-EPN query is projected into the UMAP of the integrated human spinal cord reference. Here, we used the first 30 principle components for calculation of transfer anchors and filtering was turned off. During UMAP projection, the PCA was chosen as the reference reduction and UMAP as the reduction model. Afterwards, query and reference were merged based on reference PCA and newly generated query PCA to generate a new UMAP.

For detailed analysis of SP-EPN and ependymal cells, the same procedure was conducted on the isolated ependymal cluster of the data set described above. For Pearson correlation analysis, the isolated ependymal cells and SP-EPN data were log-normalized and mean centered before together. The 8,000 most variable genes were selected and gene expression averaged for each cell population and SP-EPN subtypes. Pearson correlation was calculated using stats::cor.

Histopathological analysis

Tumor samples were histomorphologically assessed in a blinded manner by at least two experienced institutional neuropathologists based on H&E-stained tumor sections.

Statistics

Differences between clinical and biological characteristics of SP-EPN subtypes were assessed using the appropriate test as indicated in the respective figure legends. P-values <0.05 were considered statistically significant.

Illustrations

Illustrations were partly generated using BioRender.

Data availability

Methylation, RNA sequencing and whole exome sequencing data are deposited at GEO repository GSE242994. Processed RNA and whole exome sequencing data are available in Supplementary table 3-8.

Supplemental references

1. Capper D, Jones DTW, Sill M, et al. DNA methylation-based classification of central nervous system tumours. *Nature*. 2018;555(7697):469-474. doi:10.1038/nature26000

2. Tian Y, Morris TJ, Webster AP, et al. ChAMP: updated methylation analysis pipeline for Illumina BeadChips. *Bioinformatics*. 2017;33(24):3982-3984. doi:10.1093/bioinformatics/btx513

3. Hovestadt V, Zapatka M. conumee: Enhanced copy-number variation analysis using Illumina DNA methylation arrays. Published online 2023. doi:10.18129/B9.bioc.conumee

4. Mermel CH, Schumacher SE, Hill B, Meyerson ML, Beroukhim R, Getz G. GISTIC2.0 facilitates sensitive and confident localization of the targets of focal somatic copy-number alteration in human cancers. *Genome Biology*. 2011;12(4):R41. doi:10.1186/gb-2011-12-4-r41

5. Supek F, Bošnjak M, Škunca N, Šmuc T. REVIGO summarizes and visualizes long lists of gene ontology terms. *PLoS One*. 2011;6(7):e21800. doi:10.1371/journal.pone.0021800

6. Bockmayr M, Harnisch K, Pohl LC, et al. Comprehensive profiling of myxopapillary ependymomas identifies a distinct molecular subtype with relapsing disease. *Neuro-Oncology*. 2022;24(10):1689-1699. doi:10.1093/neuonc/noac088

7. Pajtler KW, Witt H, Sill M, et al. Molecular Classification of Ependymal Tumors across All CNS Compartments, Histopathological Grades, and Age Groups. *Cancer Cell*. 2015;27(5):728-743. doi:10.1016/j.ccell.2015.04.002

8. Love MI, Huber W, Anders S. Moderated estimation of fold change and dispersion for RNA-seq data with DESeq2. *Genome Biol*. 2014;15(12):550. doi:10.1186/s13059-014-0550-8

9. Wilkerson MD, Hayes DN. ConsensusClusterPlus: a class discovery tool with confidence assessments and item tracking. *Bioinformatics*. 2010;26(12):1572-1573. doi:10.1093/bioinformatics/btq170

10. Gu Z, Eils R, Schlesner M. Complex heatmaps reveal patterns and correlations in multidimensional genomic data. *Bioinformatics*. 2016;32(18):2847-2849. doi:10.1093/bioinformatics/btw313

11. Gu Z, Hübschmann D. Make Interactive Complex Heatmaps in R. *Bioinformatics*. 2022;38(5):1460-1462. doi:10.1093/bioinformatics/btab806

12. Blighe K. EnhancedVolcano: publication-ready volcano plots with enhanced colouring and labeling. Published online July 28, 2023. Accessed July 30, 2023. https://github.com/kevinblighe/EnhancedVolcano

13. Subramanian A, Tamayo P, Mootha VK, et al. Gene set enrichment analysis: a knowledge-based approach for interpreting genome-wide expression profiles. *Proc Natl Acad Sci U S A*. 2005;102(43):15545-15550. doi:10.1073/pnas.0506580102

14. Yu G, He QY. ReactomePA: an R/Bioconductor package for reactome pathway analysis and visualization. *Mol BioSyst*. 2016;12(2):477-479. doi:10.1039/C5MB00663E

15. Bockmayr M, Klauschen F, Maire CL, et al. Immunologic Profiling of Mutational and Transcriptional Subgroups in Pediatric and Adult High-Grade Gliomas. *Cancer Immunology Research*. 2019;7(9):1401-1411. doi:10.1158/2326-6066.CIR-18-0939

16. Hidalgo MR, Cubuk C, Amadoz A, Salavert F, Carbonell-Caballero J, Dopazo J. High throughput estimation of functional cell activities reveals disease mechanisms and predicts relevant clinical outcomes. *Oncotarget*. 2017;8(3):5160-5178. doi:10.18632/oncotarget.14107

17. Yadav A, Matson KJE, Li L, et al. A cellular taxonomy of the adult human spinal cord. *Neuron*. 2023;111(3):328-344.e7. doi:10.1016/j.neuron.2023.01.007

18. Zhang Q, Wu X, Fan Y, et al. Single-cell analysis reveals dynamic changes of neural cells in developing human spinal cord. *EMBO Reports*. 2021;22(11):e52728. doi:10.15252/embr.202152728

19. Hao Y, Hao S, Andersen-Nissen E, et al. Integrated analysis of multimodal single-cell data. *Cell*. 2021;184(13):3573-3587.e29. doi:10.1016/j.cell.2021.04.048

20. Tirosh I, Izar B, Prakadan SM, et al. Dissecting the multicellular ecosystem of metastatic melanoma by single-cell RNA-seq. *Science*. 2016;352(6282):189-196. doi:10.1126/science.aad0501

21. Li X, Andrusivova Z, Czarnewski P, et al. Profiling spatiotemporal gene expression of the developing human spinal cord and implications for ependymoma origin. *Nat Neurosci*. 2023;26(5):891-901. doi:10.1038/s41593-023-01312-9

22. Stuart T, Butler A, Hoffman P, et al. Comprehensive Integration of Single-Cell Data. *Cell*. 2019;177(7):1888-1902.e21. doi:10.1016/j.cell.2019.05.031
